# Design and Construction of Hybrid Microcapsules with Higher‐Order Structure and Multiple Functions

**DOI:** 10.1002/advs.201700460

**Published:** 2017-12-27

**Authors:** Guangyu Wu, Lei Wang, Pei Zhou, Ping Wen, Chao Ma, Xin Huang, Yudong Huang

**Affiliations:** ^1^ MIIT Key Laboratory of Critical Materials Technology for New Energy Conversion and Storage State Key Laboratory of Urban Water Resource and Environment School of Chemistry and Chemical Engineering Harbin Institute of Technology Harbin 150001 China

**Keywords:** higher‐order structures, hybrid microcapsules, interfacial and internal catalysis, self‐rehydration behaviors

## Abstract

The construction of inorganic‐protein hybrid microcapsules by using bovine serum albumin, metal ion clusters, and poly (*N*‐isopropylacrylamide) nanoconjugates as building blocks is presented. These microcapsules have robust membranes, which can keep their spherical morphology. They support interfacial catalytic activity by the ion clusters on their surface, and can be used as a platform to immobilize enzyme on the interface of oil/water to increase the diversity and efficiency of catalysis. These microcapsules also act as a container to make materials away from bacteria when existing silver clusters on the membrane. The obtained results highlight the construction of these microcompartments. These novel microcompartments can provide some new opportunities in bottom‐up synthetic biology, bioinspired microstorage/ microreactor, and drug/gene delivery in the future.

Recently, the design and construction of integrated chemical ensembles for mimicking key aspects of biological systems attract considerable interest in the field of drug delivery, biosensing, catalysis, and synthetic protocellular systems among the different research communities.^1^, ^2^, ^3^, ^4^ A range of synthetic microcompartments were widely reported based on using various of materials as building blocks including fatty acids (liposomes),^5^ amphiphilic polymers (polymersomes),^6^, ^7^, ^8^, ^9^, ^10^, ^11^ inorganic colloidal particles (colloidosomes),^12^, ^13^, ^14^, ^15^ and protein–polymer conjugates (proteinosomes).^16^, ^17^ Among these studies, we have paid special interest on the construction of proteinosomes based on the interfacial assembly of globular protein–polymer amphiphilic nanoconjugates via Pickering emulsion techniques. By designing and controlling the chemistry of the protein–polymer building blocks, the generated proteinosomes show a promising application toward synthetic ensembles capable of gene‐directed protein synthesis, programed release of the encapsulates, membrane‐mediated tandem catalysis, multicompartmentalization, enzyme‐mediated membrane fusion, and predatory behavior, etc.^18^ Given the flexible and ultrathin membrane of the proteinosome, to enhance the mechanical behaviors of the membrane for the further application in certain harsh environments is still a considerable challenge.

It has been well recognized that with proper design the properties of the composites could often go far beyond that achieved with single materials. Composite technology could combine multiple materials differing significantly in properties. The produced composite material with characteristics different from the constituents has progressed rapidly. In this regard, polymer/inorganic nanocomposite particles with various morphologies are by far the most common and most studied system. The combination of organic polymers with inorganic materials in a competitive and promising way can remarkably alter the practical performance like chemical, mechanical, optical, electrical, rheological, and surface properties.^19^, ^20^, ^21^ Therefore, the incorporation of inorganic nanoparticles into the membrane can endow the generated proteinosomes with some advanced inorganic nanoparticle functionalities (e.g., luminescent, magnetic, and catalytic properties).^22^, ^23^ Santhosh et al. reported fluorescence nanoclusters (NCs) using human serum albumin stabilized gold for sensitive and reliable detection of free bilirubin in blood serum samples.^24^ Guo and Irudayaraj synthesized a water‐soluble, stable, fluorescent Ag cluster via a facile green method using denatured bovine serum albumin (dBSA) as a stabilizing agent. The dBSA with its 35 free cysteine residues could contribute to polyvalent interactions with the Ag clusters and serve as effective stabilizing agents for these clusters. The as‐prepared Ag clusters were used in the detection of Hg^2+^ with high sensitivity and selectivity.^25^ However, so far, the direct construction of the higher‐order microcapsules by using the proteins/inorganic nanoparticles/polymers composite as building blocks is still rare.

Herein, we present a design and preparation of a new type of hybrid microcapsules (HPs) based on Pickering emulsion technique by using composites of protein (BSA), inorganic metal clusters (AuNCs or AgNCs), and polymer (poly (*N*‐isopropylacrylamides, PNIPAAm) as building blocks. The incorporation of the AuNCs or AgNCs into the membrane allowed the constructed HPs to show additional virtues compared with that of without metal ion hybrid capsules, including photoluminescence, self‐rehydration, enhanced antibacterial effect, and enhanced interfacial catalysis (**Scheme** **1**).

**Scheme 1:**
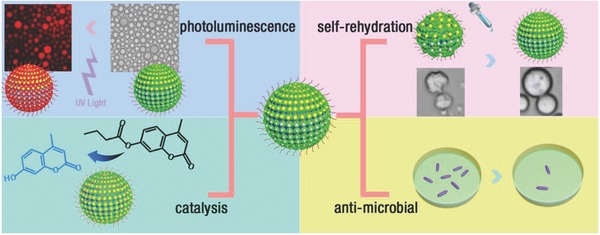
Schematic illustration showing the construction of hybrid microcapsules with different functions.

To generate the hybrid building block BSA–Au nanoclusters–PNIPAAm, first, high‐fluorescent BSA–AuNCs were synthesized,^26^ by controlled reduction of Au ions stabilized by BSA as a reducing agent. From high‐resolution transmission electron microscopy (HRTEM), the size and morphology of the obtained BSA–AuNCs were observed with an average size of AuNCs around 1.10 nm (Figure S1, Supporting Information). In comparison with the molecular weight of BSA–AuNCs and BSA studied by the Matrix‐assisted laser desorption/ionization‐time of‐flight (MALDI‐TOF) mass spectrometry (Figure S2, Supporting Information), there were 25 atoms of Au in one BSA (BSA–AuNC_25_). Moreover, circular dichroism (CD) indicated that the conjugation of AuNCs onto BSA showed very little effect on the disturbance of the BSA structure (Figure S3 and Table S1, Supporting Information).

Then by mixing the BSA–AuNC_25_ with PNIPAAm (*M*_n_ = 30 000 g mol^−1^, monomer repeat units = 259; Figures S4–S6, Supporting Information), the hybrid building block BSA–AuNC_25_–PNIPAAm was obtained by the covalent binding between the trithiol group and the AuNCs. From dynamic light scattering (DLS) study, BSA–AuNC_25_–PNIPAAm was synthesized successfully (Figure S7, Supporting Information). Also from the UV spectra calculation, on average, there were 1.20 conjugated PNIPAAm per BSA–AuNC_25_ (Figure S8 and Table S2, Supporting Information). Using Rhodamine B isothiocyanate‐labeled BSA (RITC‐BSA) as the control, the fluorescence signal preserves much more for the dots than RITC‐BSA upon prolonged continuous excitation (Figures S9 and S10, Supporting Information), indicating outstanding photostability of the BSA–AuNC_25_–PNIPAAm. The fluorescent intensity of BSA–AuNC_25_–PNIPAAm (1 mg mL^−1^) may lose 34.3% after 40 min continuous excitation, while RITC‐BSA may lose 90% intensity after 20 min continuous excitation.

When mixing an aqueous solution of BSA–AuNC_25_–PNIPAAm (10 mg mL^−1^) with 2‐ethyl‐1‐hexanol at an aqueous/oil volume fraction of 0.06, it produced well dispersion of hybrid microcapsules (HPs‐Au). The hollow structure of HPs‐Au with the diameters in the range of 7–19 µm was obtained (**Figure** **1**a). The hollow architecture is clearly visible in optical images after drying HPs, which shows a shrink but intact structure (Figure 1b). In addition, by loading a fluorescence‐isothiocyanate‐labeled dextran (FITC‐Dextran, *M*_W_ = 500 kDa) inside the HPs‐Au, the corresponding fluorescence microscopy images suggested that the formed HPs‐Au were in the form of water‐in‐oil emulsion (Figure 1c). For the constructed HPs‐Au, given the Au_25_ clusters in the membrane of HPs‐Au, this will endow the generated HPs with photoluminescence behavior. As shown in Figure 1d, the HPs‐Au presented strong red fluorescence. Additionally, the average diameter of the HPs‐Au could be also systematically controlled between 7 and 60 µm (Figure 1e; Figure S11, Supporting Information) by changing the concentration of the BSA–AuNC_25_–PNIPAAm conjugate from 0.2 to 8.0 mg mL^−1^ at an aqueous/oil volume fraction of 0.06. Moreover, the generated HPs‐Au were also confirmed by scanning electron microscopy (SEM) and HRTEM (Figure 1f,g), which clearly demonstrated the formed hollow capsule structure. The corresponding elemental mapping analysis and line profile in the HRTEM image (Figure 1h–j; Figure S12, Supporting Information) also demonstrated the homogeneous distribution of C, N, O, and S (the characteristic elements in the BSA and PNIPAAm), as well as the homogeneous distribution of Au (the characteristic elements in gold clusters) in the membrane of HPs‐Au.

**Figure 1:**
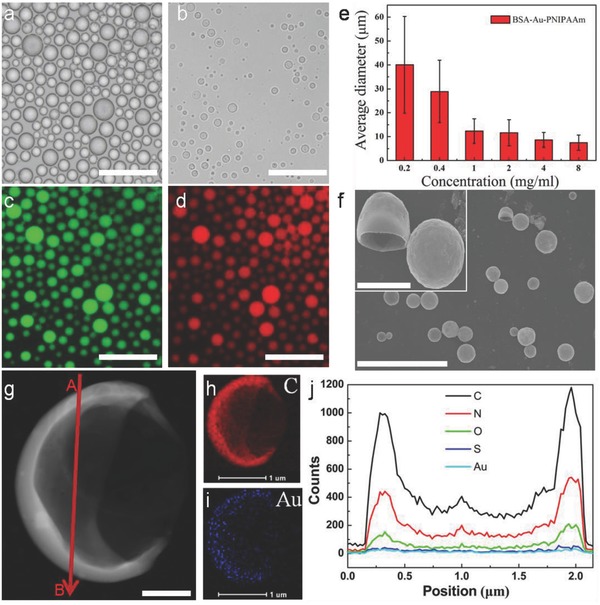
Optical microscopy images of a) HPs‐Au in oil phase and b) after partial drying in air. c,d) Fluorescence microscopy images of HPs‐Au in water‐in‐oil solution. e) Plot showing mean size (bars) and standard deviation (lines on bars) of HPs‐Au dispersed in oil and prepared at different concentrations. f) SEM showing a hollow and stabilized structure. g) HRTEM showing continuous and robust HPs‐Au membranes. h,i) The C and Au elemental distribution. j) Energy dispersive spectroscopy (EDS) line profile analysis of C, N, O, S, and Au elemental distribution taken from A to B in panel (g). Scale bars in panels (a)–(d) are 50 µm; in panel (f) is 20 µm, the inset is 3 µm; in panel (g) is 500 nm; and in panels (h) and (i) are 1 µm, respectively.

Since the ion clusters were in the membrane of HPs‐Au, this allowed the HPs‐Au to withstand complete dehydration (**Figure** **2**a). The deflated microcapsules produced on drying could be reinflated to the spherical morphology within 120 s by adding water (Figure 2b; Movie S1, Supporting Information). In contrast, collapsed BSA–NH_2_/PNIPAAm proteinosomes could not recover the original spherical structure,[[qv: 18b]] indicating that ion clusters on the surface of microcompartments considerably increased the robustness of the microarchitecture. For example, a single HP‐Au, whose original diameter size is 14.3 µm, dried to 9.2 µm after 10 h. However, the size increases to 14.1 µm after 120 s (the restoration ratio backed to 98.4%, Figure 2c). And proteinosomes could not have self‐rehydration behavior (Figure S13, Supporting Information). After transferring HPs‐Au from water‐in‐oil (w/o) and water‐in‐water (w/w) the microcapsules in aqueous solution could also keep this self‐rehydration behavior (Figures S14 and S15, Supporting Information).

**Figure 2:**
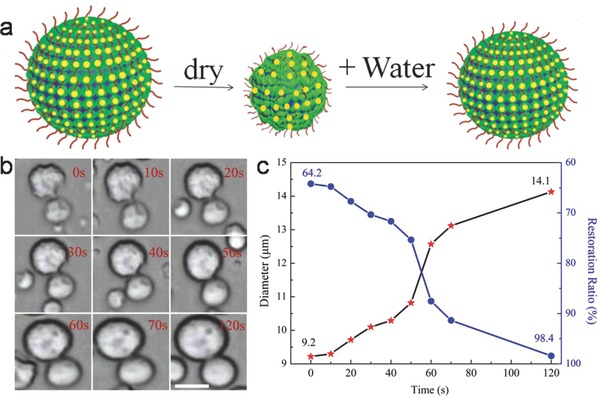
a) Schematic illustration showing self‐rehydration behavior of HPs‐Au. b) The corresponding time‐dependent optical microscopy images of single HPs‐Au by adding water during 0–120 s; scale bar, 10 µm. c) The changes of diameter and restoration ratio during the process of self‐rehydration.

Given that the fabricated HPs where the building block protein could be changed to other enzymes such as lipase and then enzyme was immobilized on the comprised membrane, this brought more functions to the constructed HPs‐Au (**Figure** **3**a,b). The lipase was labeled by a green fluorescence dye (FITC). The corresponding fluorescence microscopy images showed the presence of green fluorescence indicating that the lipase was located on the membrane of microcapsules (Figure 3c), and the red fluorescence from the Au nanocluster in the membrane (Figure 3d).

**Figure 3:**
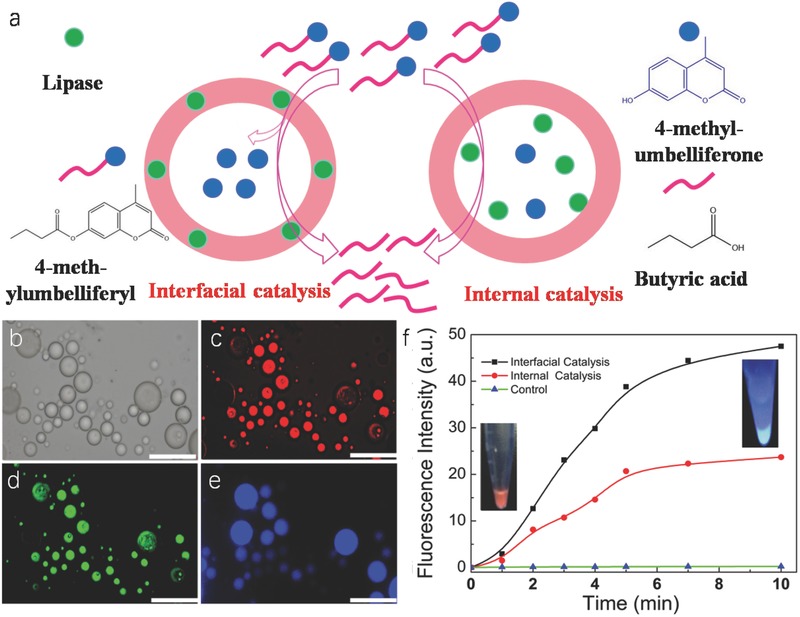
a) Schematic representation of interfacial and internal catalysis. b) Optical microscopy image in oil phase. c–e) Fluorescence microscopy images of HPs‐Au and lipase–Au–PNIPAAm HPs in aqueous solution before and after interfacial catalysis. The blue fluorescence originates from 4‐methylumbelliferone B; scale bars: 50 µm. f) The plotting of fluorescence intensity of produced 4‐methylumbelliferone at 450 nm against time by interfacial, internal catalysis, and control experiment (without lipase).

Lipases are enzymes that catalyze the hydrolysis of esters at the oil–water interface and they also have a great potential for commercial applications due to their stability, selectivity, and broad substrate specificity.^27^ In this work, the lipase‐based catalytic reaction (substrate: 4‐methylumbelliferyl butyrate) was monitored by fluorescence microscopy with the appearance of blue fluorescence hydrophilic product 4‐methylumbelliferone inside HPs‐Au over a period of 5 min (Figure 3e). In comparison with the control experiments by loading the lipases inside the microcapsules, the efficiency of the interfacial catalysis is triple that of internal catalysis by measuring the fluorescence intensity of 4‐methylumbelliferone at 450 nm within 10 min (Figure 3f; Figure S16, Supporting Information). This is mainly because interfacial catalysis is the direct hydrolysis of lipase at the oil–water interface, whereas the internal catalysis is due to hindrance of membrane of HPs‐Au, leading to a decrease in catalytic efficiency.^28^ Moreover, it should be mentioned that the AuNCs in the membrane of the HPs‐Au still maintained the catalytic behavior, which can catalyze 1,2‐diaminobenzene into poly‐*o*‐phenylenediamine, as observed the appearance of orange color product at 450 nm (Figure S17, Supporting Information).

In addition, to further enhance the antibacterial activities of the HPs against *Escherichia coli* (8099) and *Staphylococcus aureus* (ATCC 6538), similarly, Ag nanoclusters were synthesized to replace Au nanoclusters to generate the BSA–AgNCs–PNIPAAm. From HRTEM, the size and morphology of the obtained BSA–AgNCs were observed with an average size of AgNCs around 1.44 nm (Figure S18, Supporting Information). In comparison with the molecular weight of BSA–AgNCs and BSA studied by the MALDI‐TOF (Figure S19, Supporting Information), there were 15 atoms of Ag in one BSA (BSA–AgNC_15_). Moreover, CD indicated that the conjugation of AgNCs onto BSA showed very little effect on the disturbance of the BSA structure (Figure S20 and Table S2, Supporting Information).

After mixing the BSA–AgNC_15_ with PNIPAAm, the hybrid building block BSA–AgNC_15_–PNIPAAm was obtained by the covalent binding between the trithiol group and the AuNCs. From DLS study, BSA–AgNC_15_–PNIPAAm was synthesized successfully (Figure S21, Supporting Information). Also from the UV spectra calculation, on average, there were 1.35 conjugated PNIPAAm per BSA–AgNC_15_ (Figure S22 and Table S4, Supporting Information). Using RITC‐BSA as the control, the fluorescence signal preserves much more for the dots than RITC‐BSA upon prolonged continuous excitation (Figures S23 and S24, Supporting Information), indicating outstanding photostability of the BSA–AgNC_15_–PNIPAAm. When mixing an aqueous solution of BSA–AgNC_15_–PNIPAAm (10 mg mL^−1^) with 2‐ethyl‐1‐hexanol at an aqueous/oil volume fraction of 0.06, it produced well dispersion of hybrid microcapsules (HPs‐Ag). The hollow structure HPs‐Ag with the diameters in the range of 5–22 µm (Figure S25a, Supporting Information) was obtained. The hollow architecture is clearly visible in optical images after drying HPs, which shows a shrink but intact structure (Figure S25b, Supporting Information). In addition, by loading FITC‐Dextran (*M*_W_ = 500 kDa) inside the HPs‐Ag, the corresponding fluorescence microscopy images suggested that the formed HPs‐Ag were in the form of water‐in‐oil emulsion (Figure S25c, Supporting Information). For the constructed HPs‐Ag, given the Ag_15_ clusters in the membrane of HPs‐Ag, this will endow the generated HPs with photoluminescence behavior. As shown in Figure S25d (Supporting Information), the HPs‐Ag presented strong red fluorescence. Additionally, the average diameter of the HPs‐Au could also be systematically controlled between 12 and 67 µm (Figures S25e and S26, Supporting Information) by changing the concentration of the BSA–AgNC_15_–PNIPAAm conjugate from 0.2 to 8.0 mg mL^−1^ at an aqueous/oil volume fraction of 0.06. Moreover, the generated HPs‐Ag were also confirmed by SEM (Figure S27, Supporting Information), which clearly demonstrated the formed hollow capsule structure. The corresponding elemental mapping analysis and line profile in the HRTEM image (Figure S28, Supporting Information) also demonstrated the homogeneous distribution of C, N, O, and S (the characteristic elements in the BSA and PNIPAAm), as well as the homogeneous distribution of Ag (the characteristic elements in silver clusters) in the membrane of HPs‐Ag. Since the ion clusters were in the membrane of HPs‐Ag, this allowed the HPs‐Ag to withstand complete dehydration. The deflated microcapsules produced on drying could be reinflated to the spherical morphology within 10 s by adding water (Figures S29–S31 and Movie S2, Supporting Information). Bacterial growth rates were determined by measuring the optical density at 600 nm (OD600) based on the turbidity of the cell suspension. Figure S32a,b (Supporting Information) shows the antibacterial activities of the protein‐based microcapsules without metal ion, HPs‐Au and HPs‐Ag, respectively. One can see that both the HPs‐Au and HPs‐Ag could obviously slow the growth of *E. coli* and *S. aureus* due to the fact that the inorganic parts, especially silver, could inhibit their growth.

In conclusion, a direct construction of the hybrid microcapsules has been developed by using the proteins/inorganic metal clusters/PNIPAAm as building blocks with robust membranes for supporting interfacial catalytic activity and acting as a platform to immobilize enzyme on the interface of oil/water to increase the diversity and efficiency of catalysis. These microcapsules help the incorporation of the AuNCs or AgNCs into the membrane with additional virtues of the photoluminescence, self‐rehydration, enhanced antibacterial effect, and interfacial catalysis. The hybrid microcapsules described in this work extend the concepts of HPs' construction‐based microcompartments for providing some new opportunities in bottom‐up synthetic biology, bioinspired microstorage/microreactor as well as drug/gene delivery.

## Conflict of Interest

The authors declare no conflict of interest.

## Supporting information

Supplementary

Supplementary

Supplementary
