# Supplementary material for: Design and Construction of Hybrid Microcapsules with Higher‐Order Structure and Multiple Functions
Source: Adv Sci (Weinh). 2017 Dec 27;5(2):1700460. doi: 10.1002/advs.201700460 (PMC5827534; doi:10.1002/advs.201700460)
Supplement: Supplementary file 1 — Supplementary [file ADVS-5-1700460-s002.pdf]

Copyright WILEY-VCH Verlag GmbH & Co. KGaA, 69469 Weinheim, Germany, 2017.

Supporting Information

for *Adv. Sci.,* DOI: 10.1002/advs.201700460

Design and Construction of Hybrid Microcapsules with Higher-Order Structure and Multiple Functions

Guangyu Wu, Lei Wang, Pei Zhou, Ping Wen, Chao Ma, Xin Huang,* and Yudong Huang**

Supporting Information

Design and Construction of Hybrid Microcapsules with Higher-Order Structure and Multiple Functions

Guangyu Wu, Lei Wang, Pei Zhou, Ping Wen, Chao Ma, Xin Huang,* and Yudong Huang**

Supporting Information

Design and Construction of Hybrid Microcapsules with Higher-Order Structure and Multiple Functions

Guangyu Wu, Lei Wang, Pei Zhou, Ping Wen, Chao Ma, Xin Huang,* and Yudong Huang**

Dr. G. Wu, Dr. L. Wang, P. Zhou, P. Wen, C. Ma, Prof. X. Huang, Prof. Y. Huang,

MIIT Key Laboratory of Critical Materials Technology for New Energy Conversion and Storage

State Key Laboratory of Urban Water Resource and Environment

School of Chemistry and Chemical Engineering

Harbin Institute of Technology

Harbin 150001, China.

E-mail: leiwang_chem@hit.edu.cn; xinhuang@hit.edu.cn; huangyd@hit.edu.cn

Chemicals and materials:1-Propanethiol (Aladdin, 99%), carbon disulfide (Sigma, 99%), potassium ferricyanide ($K_3Fe(CN)_6$, Aladdin, 99%), 4,4’-azobis (4-cyanovaleric acid) (ACVA, Sigma, 98%), 2,2'-Dithiodipyridine (Aladdin, 98%), 2-Mercaptoethanol (Sigma, 99%). N’-dicyclohexylcarbodiimide (DCC, Fluka, 99 %), 4-(dimethylamino)pyridine (DMAP, Aldrich 99%), Tris(2,2-bipyridine) dichlororuthenium(II) hexahydrate (Aladdin, 98%), N-isopropylacrylamide (NIPAAm, Sigma, 98 %) was recrystallized twice in hexane and toluene prior to use. 2-ethyl-1-hexanol (Sigma, ≥98%), PEG-bis (N-succinimidyl succinate (Sigma, 98 %), Chloroauricacid ($HAuCl_4 \cdot H_2O$, Energy Chemical, 98%), silver nitrate ($AgNO_3$, Energy Chemical, 99.8%), sodiumhydroxide (NaOH) and sodium borohydride ($NaBH_4$) were purchased from Guangfu Technology Development Co. Ltd. (Tianjin, China). Albumin from bovine serum (BSA, isoelectric point = 4.6) (Sigma, ≥98%) were used as received without further purification

Characterization methods: ^{1}H-NMR spectra were recorded on Bruker Advance-400 MHz spectrometer with $CDCl_3$ as solvents at room temperature. Chemical shifts (δ) were expressed in ppm and coupling constants (J) in Hertz. Transmission electron microscopy (TEM) analysis was undertaken on a JEM-1400 and TEM using a LaB6 filament at 120 kV in bright field mode. Samples were prepared by adding one drop of microcapsule solution (0.1 mg/mL) onto a 300 mesh, carbon film coated copper grid and the specimens were then dried in vacuum for one day. SEM images were obtained on a HITACHI UHR FE-SEMSU8000 with the samples sputter-coated with

10 nm platinum. Optical and fluorescence microscopy was performed on a Leica DMI8manual inverted fluorescence microscope at 10x, 20x, 40x and 100x magnification. Fluorescence measurements were performed on a PerkinElmer spectrofluorometer (LS-55, USA). UV-Vis spectra were measured on a PerkinElmer spectrophotometer (Lambda 750S, USA). The average particle size and size distribution of building blocks (BSA-Au-PNIPAAm/BSA-Ag-PNIPAAm, 0.2 mg/mL, pH 6.8, 5.0 mM PBS buffer) were characterized by dynamic light scattering (DLS) with an ALV-5000/E DLS instrument (Malvern Instruments, UK) a fixed scattering angle of 90°, after being filtered by 0.45 μm Milli-pore filters. Circular dichroism (CD) spectra of the native and released in sulin from the capsules at 200−300 nm in PBS buffer were obtained by a CD spectropolarimeter (Jasco-715, JPN) at room temperature. The pH measurements were made with a Seven Compact meter (METTLER TOLEDO, SUI). Oscillator was employed by a VORTEX instrument (IKA, GER). Assimilated solutions were executed by pipettors (GILSON, FRA).

Sample synthesis: Synthetic routes employed for the preparation of mercaptopyridine-activated trithiol-RAFT agent 2-(pyridin-2-yldisulfanyl) ethyl 4-cyano-4-(((propylthio)carbonothioyl)thio) pentanoate and poly(N-isopropylacrylamide) (PNIPAAm, Figure S1-S3) and two types of protein-metalmaterials (BSA-Au NCs and BSA-Ag NCs).

Synthesis of 4-cyano-4-(propylsulfanylthiocarbonyl) sulfanylpentanoic acid:

Propanethiol (5.79 g, 76 mmol) was added over 10 min to a stirred suspension of sodium ethoxide (5.44g, 80mmol) in diethyl ether (150 mL) at 0 °C. The solution was then allowed to stir for 10 min prior to the addition of carbon disulfide (6.0 g, 79 mmol). Crude sodium S-propyl trithiocarbonate (6.28 g, 40 mmol) was collected by filtration, suspended in diethyl ether (100 mL) and reacted with iodine (9.88 g, 30mmol). After 2 h, the solution was filtered, washed with aqueous sodium thiosulfate, and dried over sodium sulfate. The crude bis(propylsulfanylthiocarbonyl) disulfide was then isolated by rotary evaporation. Then a solution of bis(propylsulfanylthiocarbonyl) disulfide (2.42 g, 8.8 mmol) and 4,4'-azobis(4-cyanovaleric acid) (ACVA, 4.01 g, 14.3 mmol) in ethyl acetate (200 mL) was degassed by nitrogen for 30 mins and heated to 80 ℃ for 8 h. The crude product was further purified by flash column chromatography on petroleum ether /ethyl acetate (2:1 volume ratio).

Synthesis of 2-(2-dimercaptopyridine) ethanol: A solution of 2,2'-dithiodipyridine （7.52 g, 34 mmol） and 2-mercaptoethanol (1.33 g, 17 mmol) dissolved in methanol (100 mL) at room temperature under rigorous stirring. After 3 h, the crude product was further purified by flash column chromatography on petroleum ether/ethyl acetate (3:2 volume ratio)

Synthesis of mercaptopyridine-activated trithiol-RAFT agent 2-(pyridin-2-yldisulfanyl) ethyl 4-cyano-4-(((propylthio)carbonothioyl)thio)

pentanoate: A solution of 2-(2-dimercaptopyridine) ethanol (4.01 g, 14.3 mmol) and 4-cyano-4-(propylsulfanylthiocarbonyl) sulfanylpentanoic acid (4.50 g, 37.8 mmol) in 1,4-dioxane (200 mL) was degassed by nitrogen for 30 mins. Then N,N'-dicyclohexylcarbodiimide (DCC, 6.82 g, 33.0 mmol) and 4-(dimethylamino)-pyridine (DMAP, 0.10 g, 0.82 mmol) dissolved in 1,4-dioxane (100 mL) were added slowly at room temperature under rigorous stirring. After 20 h, the reaction mixture was filtered and concentrated. The mercaptopyridine-activated trithiol-RAFT agent 2-(pyridin-2-yldisulfanyl) ethyl 4-cyano-4- (((propylthio)carbonothioyl)thio) pentanoate was obtained. ^{1}H-NMR (400 MHz, $CDCl_3$): 8.50 (1H, s), 7.73 (1H, s), 7.71 (1H, s), 7.17 (1H, s), 4.37-4.40 (2H, m), 3.30-3.34 (2H, m), 3.06-3.08 (2H, m), 2.55-2.61 (2H, m), 2.33-2.41 (2H, m), 1.88(1H, s), 1.65-1.79 (3H, t, J=56),1.01-1.04 (3H,m).

Synthesis of end-capped mercaptopyridine-activated PNIPAAm by RAFT polymerization: Mercaptopyridine-activated trithiol-RAFT agent (3 mg, 6.7 µmol), tris(2,2'-bipyridine) dichlororuthenium (II) hexahydrate (0.1 mg, 0.13 µmol), NIPAAm (900 mg, 7.97 mmol) and acetonitrile (2 mL) were added to a 10 mL of round-bottom flask. The flask was then sealed and the solution was degassed via four freeze pump-thaw cycles. The polymerization was carried out at UV-irradiation (395nm) for 36 h, and purified by three times precipitation in diethylether/hexane (2:1 volume ratio). The obtained polymer was characterized by ^{1}H-NMR spectroscopy in $CDCl_3$. The proton signal (δ, 4H. 7.17-8.50 ppm) from the pyridine at the end of the

PNIPAAm chain was clearly visible in the ^1H-NMR spectrum. The molecular weight of the obtained PNIPAAm was determined by ^1H-NMR by comparing the integral of the proton of the CH signal at δ=7.17 ppm in pyridine with that of the characteristic CH signal at δ=4.0 ppm in the repeat unit of NIPAAm ($M_n \approx 30000$ g/mol).

Synthesis of BSA-AuNCs and BSA-Ag NCs: All glasses used in the experiments were cleaned by the freshly prepared Aqua Regia (HCl: HNO_3 volume ratio = 3: 1), and rinsed thoroughly in water prior to use. BSA-AuNCs (1:1) were prepared according to previous report with modification.[1,2] Briefly, BSA (250 mg) was dissolved in deionized water (5 mL) under stirring. Chloroauric acid (1.25 mmol) was drop wisely added to the solution, after30min incubation in 37°C, the pH of this mixture was adjusted to 12 by adding NaOH (0.125 mol) and the bottle was incubated in 37 °C oven for 12h.Then the solution was dialyzed (dialysis tubing 8-14 kDa MWCO) extensively against Milli-Q water and freeze-dried on a Freeze-Dry System (P ≈ 10^{-4} bar for 24 h) and stored at -20 ^{o}C.

For the synthesis of BSA-AgNCs,[3] 10 mM silver nitrate (5 mL, $AgNO_3$) solution was added to 250 mg BSA powder in 5 mL distilled water with vigorous stirring at room temperature. Then 0.3 mL NaOH (1 M) was added followed by 10 mM $NaBH_4$ solution drop-wise until the solution turns from colourless to reddish brown, indicating the formation of various amounts of clusters. Then the solution was dialyzed (dialysis tubing 8-14 kDa MWCO) extensively against Milli-Q water and

freeze-dried on a Freeze-Dry System (P ≈ 10^{-4} bar for 24 h) and stored at -20 ^{o}C.

Synthesis of BSA-Au NCs-PNIPAAm and BSA-Ag NCs-PNIPAAm nanoconjugates: Mercaptopyridine-activated PNIPAAm was added to a stirred solution of BSA-Au NCs or BSA-Ag NCs (10 mg in 5 mL of deionized water). The mixed solution was stirred for 0.5 h, and then purified by using a centrifugal filter (MWCO 50 kDa) to remove any unreacted PNIPAAm and salts. After freeze-drying, the BSA-Au-PNIPAAm and BSA-Ag-PNIPAAm conjugates were obtained.

Preparation of hybrid microcapsules: Microcapsules were prepared by mixing an aqueous BSA-AuNCs-PNIPAAm or BSA-AgNCs-PNIPAAm solution with 2-ethyl-1-hexanol followed by shaking the mixture by hand for 10 s. The samples were prepared at a constant aqueous/oil volume fraction of 0.06. 0.06 mL of aqueous BSA-AuNCs-PNIPAAm or BSA-AgNCs-PNIPAAm (10 mg/mL, pH 8.5, sodium carbonate buffer) was mixed with 1.0 ml of the oil. The fabrication process of proteinosomes was conducted according to a previous report [4] with few modifications, and the details were shown in the following parts.

Synthesis of the cationized bovine serum albumin (BSA-NH$_2$): Cationized bovine serum albumin (BSA-NH$_2$) was synthesized by carbodiimide activated conjugation of 1, 6-diaminohexane to aspartic and glutamic acid residues on the external surface of the protein. For this, a solution of 1, 6-diaminohexane (1.5 g, 12.9 mmol) was

adjusted to pH 6.5 using 5 M HCl and added dropwise to a stirred solution of the protein (200 mg, 2.98 μmol). The coupling reaction was initiated by adding N'-ethyl-N'-(3-(dimethylamino) propyl) carbodiimide hydrochloride (EDAC, 100 mg) immediately and again (50 mg) after 5 h. The pH value was maintained at 6.5 using dilute HCl, and the solution was stirred for a further 6 h. The solution was then centrifuged to remove any precipitate, and the supernatant was dialyzed (dialysis tubing 12-14 kDa MWCO) extensively against Milli-Q water.

Synthesis of BSA-NH$_2$/PNIPAAm conjugates: End-capped mercaptothiazoline-activated PNIPAAm (10 mg in 5 mL of water) was synthesized according to our previous reported method and added to a stirred solution of BSA-NH$_2$ (10 mg in 5 mL of PBS buffer at pH 8.0). The mixed solution was stirred for 12 h, and then purified by using a centrifugal filter (MWCO 50 kDa) to remove any unreacted PNIPAAm and salts. After freeze-drying, the BSA-NH$_2$/PNIPAAm conjugate was obtained.

Preparation of proteinosomes: Proteinosomes were prepared by mixing an aqueous BSA-NH$_2$/PNIPAAm solution with 2-ethyl-1-hexanol followed by shaking the mixture by hand for 10 s. The samples were prepared at a constant aqueous/oil volume fraction (φ_w) of 0.06. Typically, 0.06 mL of aqueous BSA-NH$_2$/PNIPAAm (10 mg/mL, pH 8.5, sodium carbonate buffer) were mixed with 1.0 mL of the oil.

Transferring proteinosomes and hybrid microcapsules into aqueous solution: The

proteinosomes and hybrid microcapsules were then cross-linked in the continuous oil phase by addition of PEG-bis(N-succinimidyl succinate) (0.5 mg), which reacted with free remnant primary amine groups of BSA. Transfer of the cross-linked microcapsules into water was achieved as follows. After 3 h sedimentation, the upper clear oil layer was discarded and 1 mL of 70% ethanol was added. The proteinosomes and hybrid microcapsules were washed three times by 70% ethanol via centrifugation-disperse process, then washed by Milli-Q water to complete the phase transfer process.

Catalysis 1,2-diaminobenzene (OPD) to poly-o-phenylenediamine (POPD): 0.06 mL of aqueous BSA-AuNCs-PNIPAAm (10 mg/mL, pH 8.5, sodium carbonate) was mixed with 1.0 mL of the oil (216 mg OPD dissolved in it). After shaking the mixture by hand for 10 s, the emulsion was obtained. Every two minutes, 0.1 mL emulsion was added to 0.4 mL ethanol and used UV-vis spectrum to test the absorbance at 450 nm.

Self-rehydration: The self-rehydration of hybrid microcapsules and proteinosomes were performed under the optical microscopy directly. One droplet of the hybrid microcapsules solution was added onto the glass slide and dried in air. After 30 mins, the microcapsules could be dried completely. Then some water was added onto the glass slide, the hybrid microcapsules could self-rehydrate, while proteinosomes could not have this function.

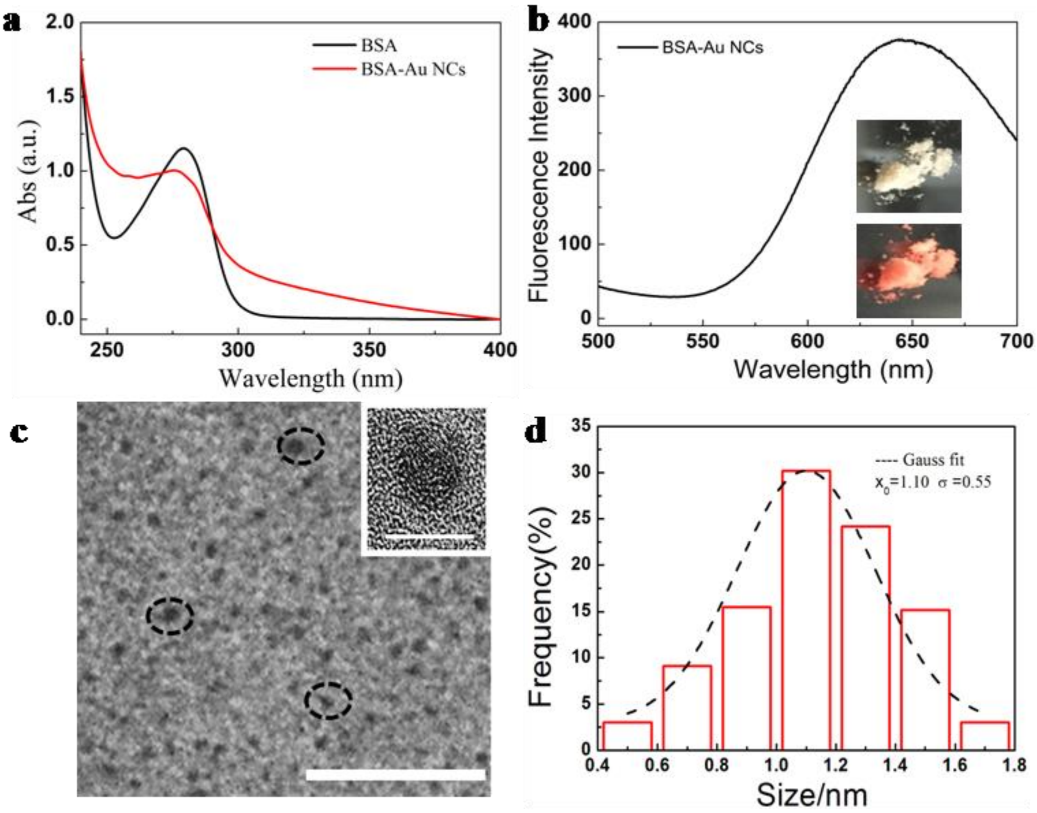

Figure S1. UV-vis absorption (a) and emission (b) spectra of the as prepared BSA-Au NCs, the inset Photographs of BSA-Au NCs powder under (top) visible and (bottom) UV light. TEM images of BSA-AuNCs (c). Particle size distributions of AuNCs (d).

The absorption spectra of BSA and BSA-AuNCs are shown in Figure S1a. The additional new absorbance seen over the 300-450 nm regions was attributed to the HOMO-LUMO electronic transition within Au clusters, suggesting the successful synthesis of the BSA-AuNCs. By using 380 nm as exciting wavelength, the obtained BSA-AuNCs showed a strong fluorescence emission at 630 nm (Figure S1b). The obtained BSA conjugated clusters showed excellent stability over a wide range of pH from 4 to 12 and could be stored in the form of powder. Moreover, from high resolution transmission electron microscopy (HRTEM), the size and morphology of the obtained BSA-AuNCs were observed. As shown in Figure S1 (c-d), the

synthesized BSA-AuNCs were well monodispersed. Since the low constrast of BSA in the TEM, only the AuNCs could be discerned with an average size of was around 1.10nm which also consistent.

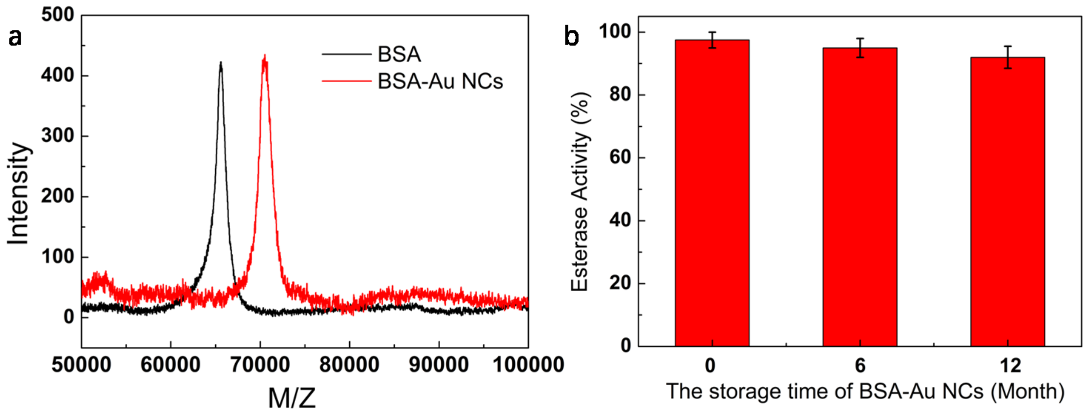

Figure S2. (a) MALDI-TOF mass spectra of BSA (black) and BSA-AuNCs (red); (b) Room temperature esterase activity of BSA-Au NCs during one year at pH 8.5 using p-nitrophenyl butylester (2.0 mM) as a substrate

From **Figure S2a**, BSA showed a broad mass peak around m/z **65.5kDa**, and the as-prepared BSA-AuNCs (red) showed a peak around m/z **70.5kDa**. From Figure S2b, BSA-Au NCs could keep stability by testing esterase activity; and the results showed that the BSA-Au NCs could be saved for about one year.

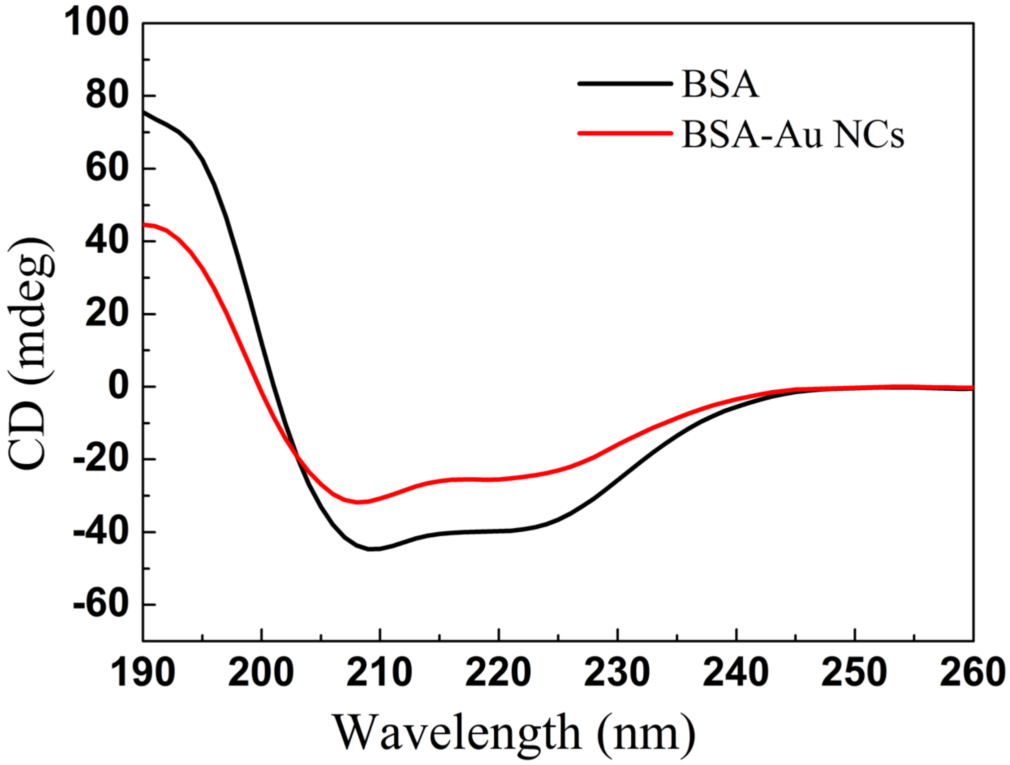

Figure S3. CD spectra for BSA and BSA-Au NCs dispersed in aqueous solution at room temperature.

Table S1. Secondary structure content in various BSA and BSA-Au NCs constructs at 25℃.

	α helix	β strand
BSA	67.29%	10.06%
BSA-Au NCs	67.16%	10.87%

In the comparison of the CD spectra of native BSA and BSA-AuNCs in the wavelength range between 190 and 260 nm, there were minimal changes in the characteristic peak intensities at 208 nm and 222 nm (the characteristics of the transitions of π–π* and n–π* of α-helical structure of BSA) for native BSA and BSA-AuNCs dispersed in water (Figure S3). The deconvolution of the CD spectra showed that the levels of α-helical secondary structure and the contents of the β-sheet for BSA and BSA-AuNCs were 67.29%, 10.06%, 67.16%and 10.87%, respectively (Table S1).

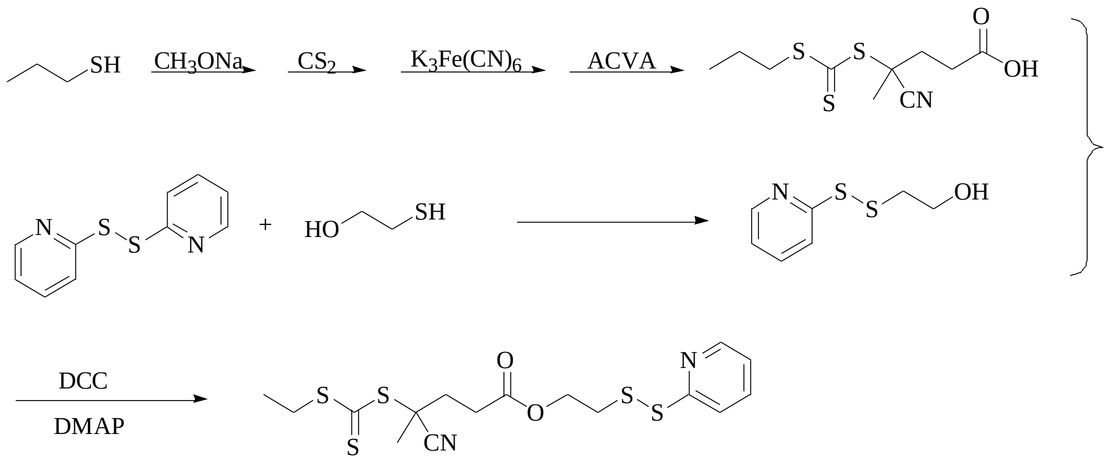

Figure S4. The general procedure for the synthesis of the mercaptopyridine-activated trithiol-RAFT agent 2-(pyridin-2-yldisulfanyl) ethyl 4-cyano-4-(((propylthio)carbonothioyl)thio) pentanoate.

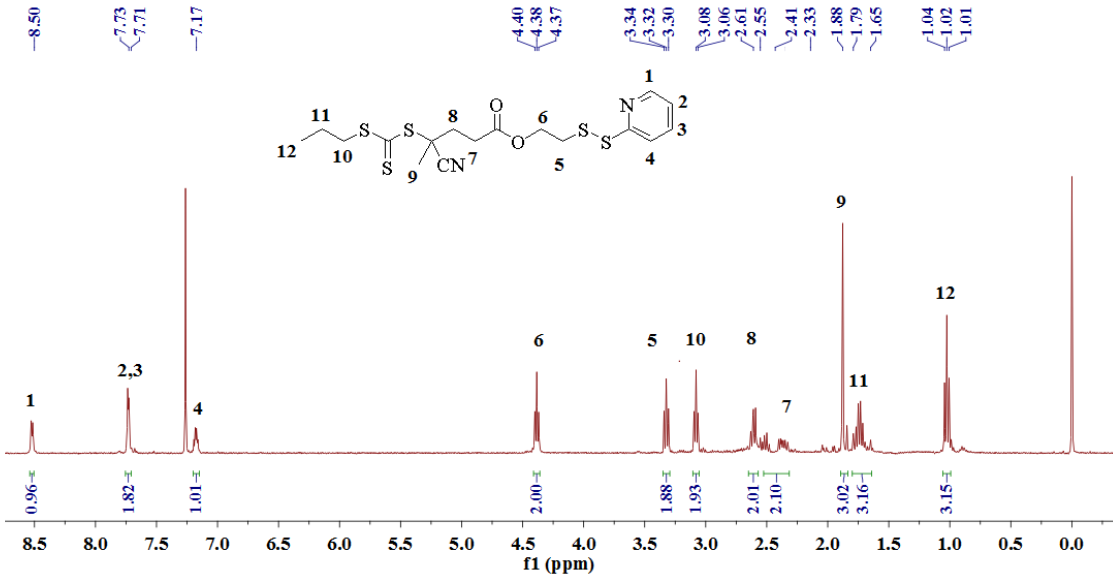

Figure S5. ^{1}H-NMR spectrum of 2-dimercaptopyridine-activated trithiol-RAFTagent 4-cyano-4-(propylsulfanylthiocarbonyl) sulfanyl pentanoic acid 2-(2-dimercaptopyridine) ethanol in $CDCl_3$.

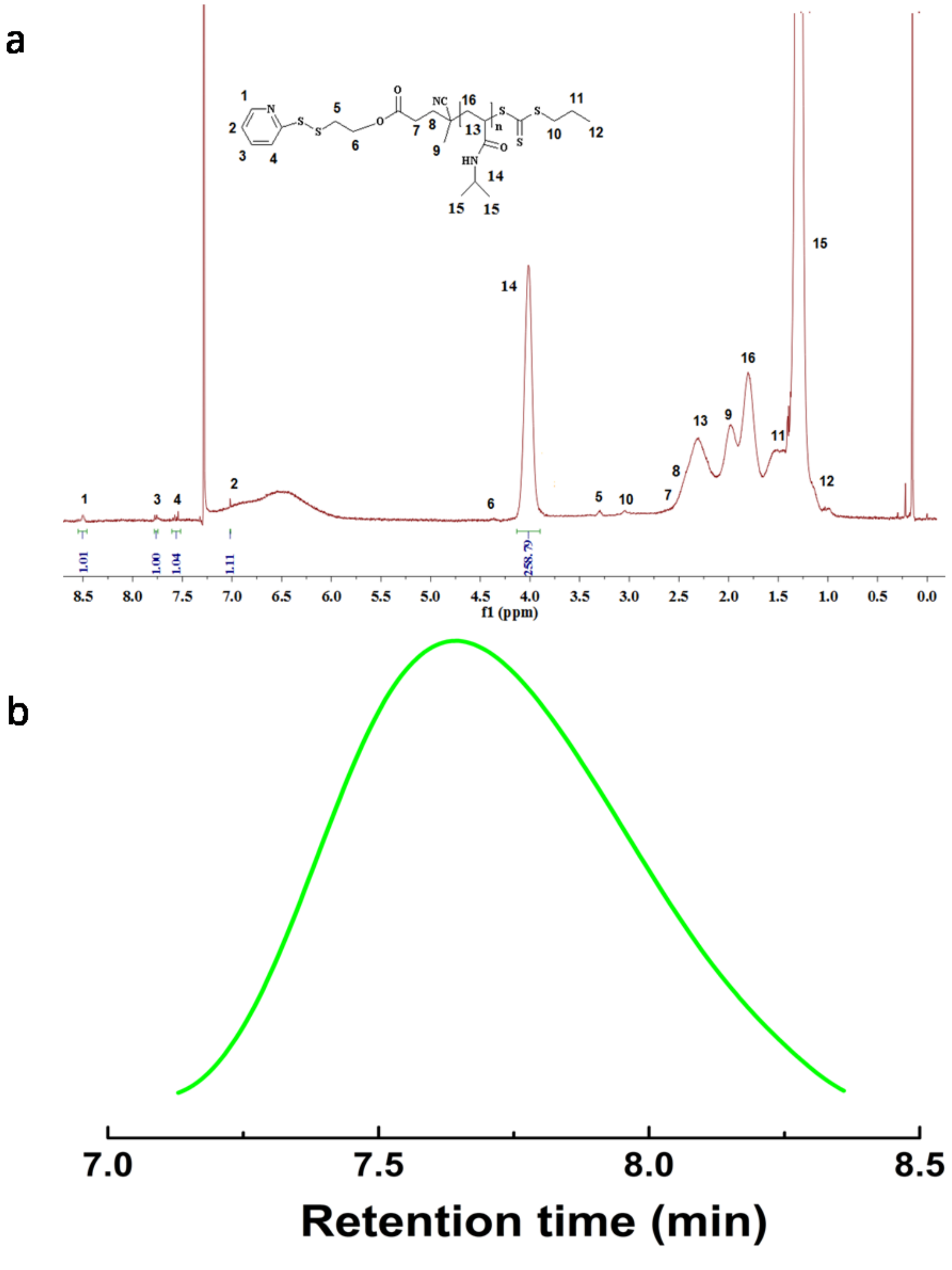

Figure S6. (a) ^{1}H-NMR spectrum of 2-dimercaptopyridine-activated trithiol-activated PNIPAAm in $CDCl_3$. (b) GPC profile of PNIPAAm. Tetrahydrofuran (THF) was used as the eluent witha PNIPAAm concentration of 1.0 mg/mL. The PDI value was 1.35.

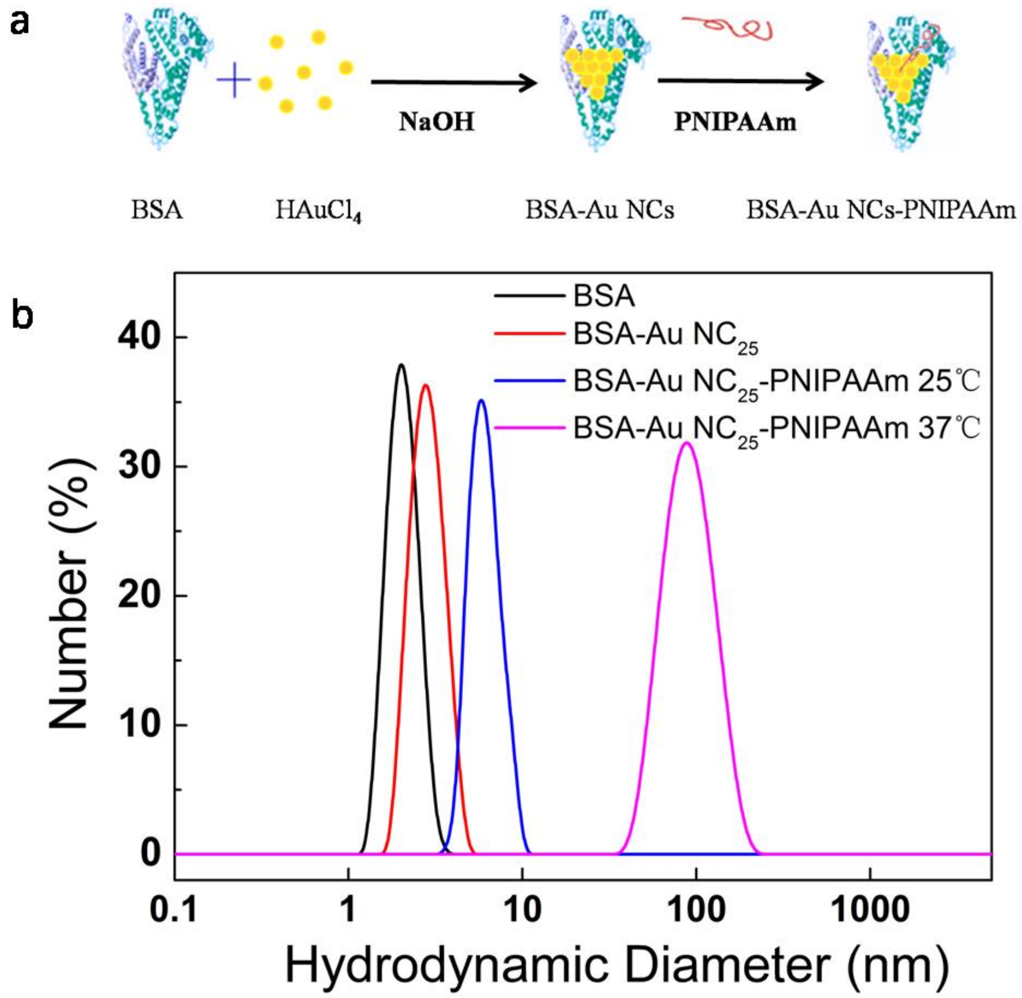

Figure S7. (a) Schematic of the Formation of BSA-Au NC25-PNIPAAm.(b)DLS profiles for aqueous BSA, BSA-AuNC$_{25}$ and BSA- Au NC$_{25}$-PNIPAAm conjugates recorded at 25℃. Profile for aqueous BSA- Au NC$_{25}$-PNIPAAm at 37℃ is also shown. The Dh values of BSA, BSA-Au NC$_{25}$ and BSA-Au NC$_{25}$-PNIPAAm conjugates recorded at 25℃ are 2.02, 3.12 and 5.62 nm, respectively. Dh value for aqueous BSA-Au NC$_{25}$-PNIPAAm at 37℃ is 89.43 nm. The PDI values of BSA, BSA-Au NC$_{25}$ and BSA-Au NC$_{25}$-PNIPAAm conjugates recorded at 25℃ are 0.835, 0.453 and 0.362, respectively. PDI value for aqueous BSA-Au NC$_{25}$-PNIPAAm at 37℃ is 0.141.

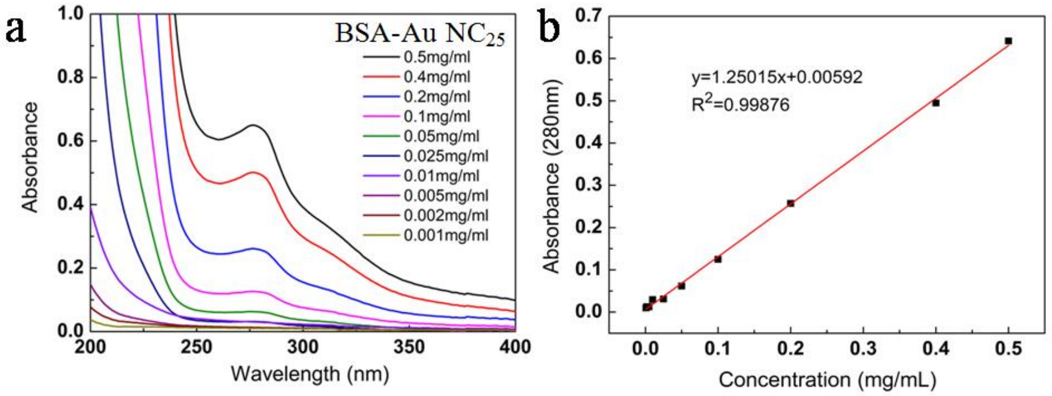

Figure S8. (a) UV-vis spectra of native BSA-AuNC$_{25}$ at different concentrations from 0.001 to 0.5mg/mL in aqueous solution. (b) Calibration curve for native BSA-AuNC$_{25}$ determined by plotting the UV-vis absorbance at 280 nm against concentration. The calibration was used to determine the number of PNIPAAm chains per protein molecule in the BSA-AuNC$_{25}$-PNIPAAm nanoconjugates (see Table S2 below)

Table S2. PNIPAAm : BSA-Au NC_{25} stoichiometry in BSA-AuNC_{25}-PNIPAAm conjugates based on UV-vis spectroscopic analysis of three different sample concentrations.

	Concentration (mg/mL)	A(280)	PNIPAAm Content （chains perBSA-AuNC_{25}-PNIPAAm）
Test 1	0.05	0.04730	1.21
Test 2	0.10	0.08991	1.16
Test 3	0.50	0.41482	1.24

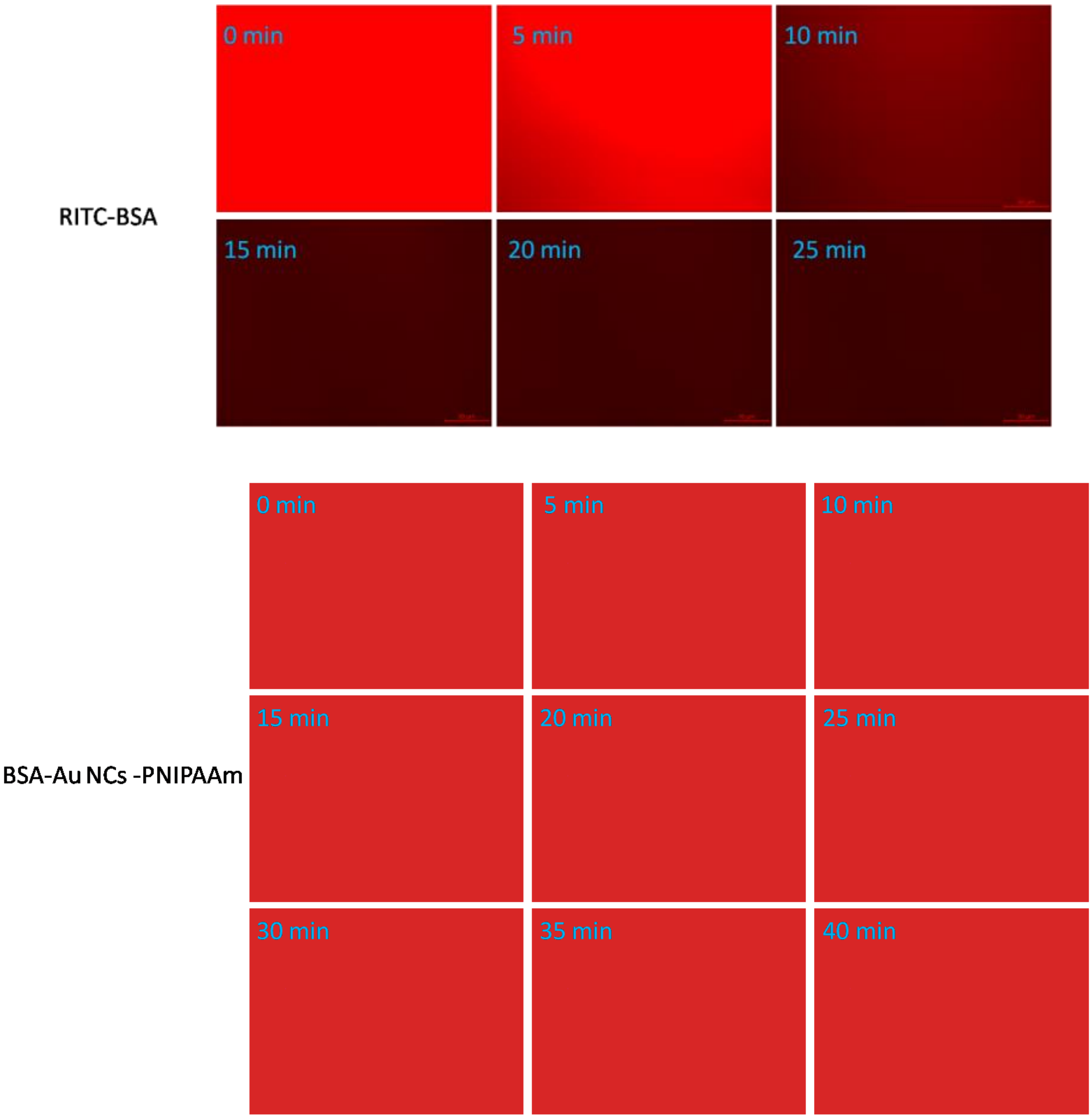

Figure S9. Photostability of BSA-AuNC$_{25}$-PNIPAAm (1 mg/mL) compared to Rhodamine B isothiocyanate labeled BSA(RITC-BSA) under continual excitation on fluorescence microscope.

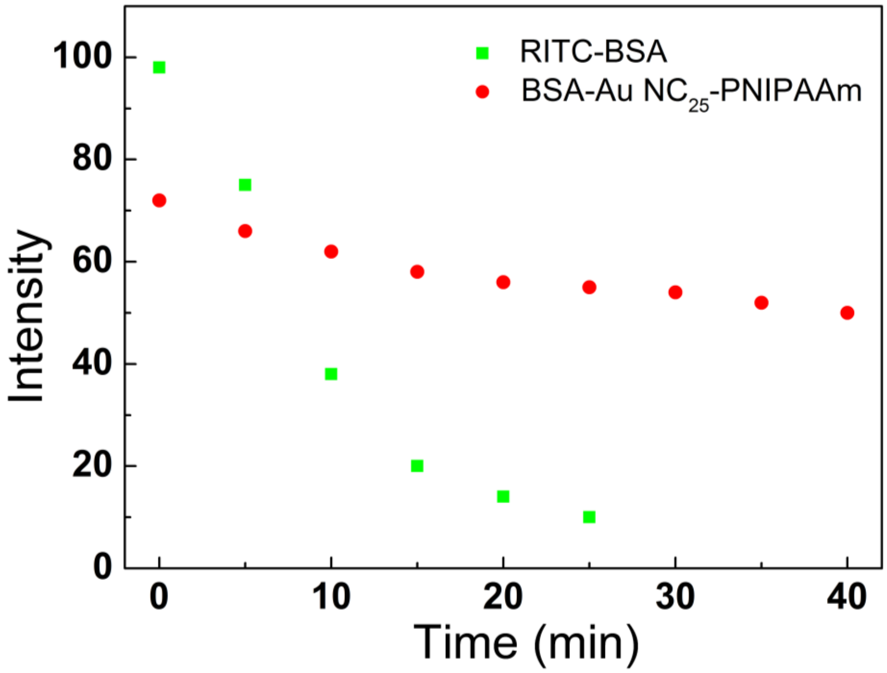

Figure S10. Data derived from Figure S9 quantified by ImageJ software.

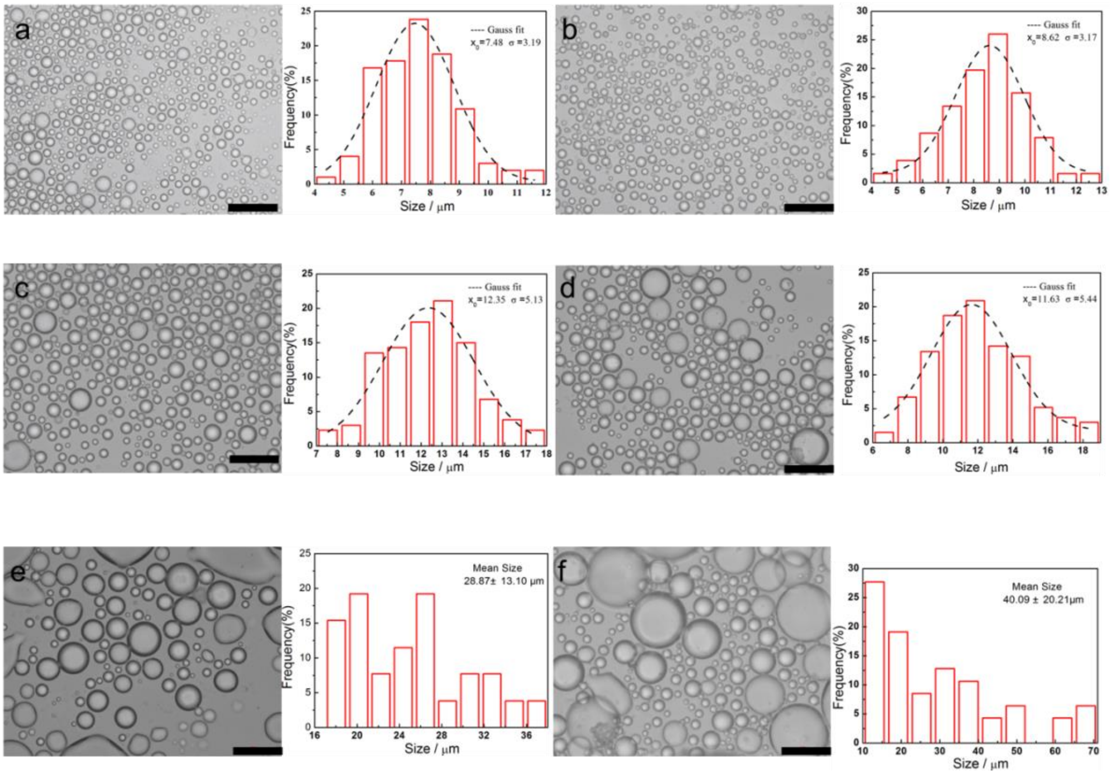

Figure S11. (a-f) Particle size distribution of hybrid proteinosomes prepared at different concentrations of BSA-AuNC$_{25}$-PNIPAAm (8.0, 4.0, 2.0, 1.0, 0.4, and 0.2 mg/mL, respectively). Median values and standard deviations were calculated by fitting Gaussians to the histograms in a-d. All scare bars are 50μm.

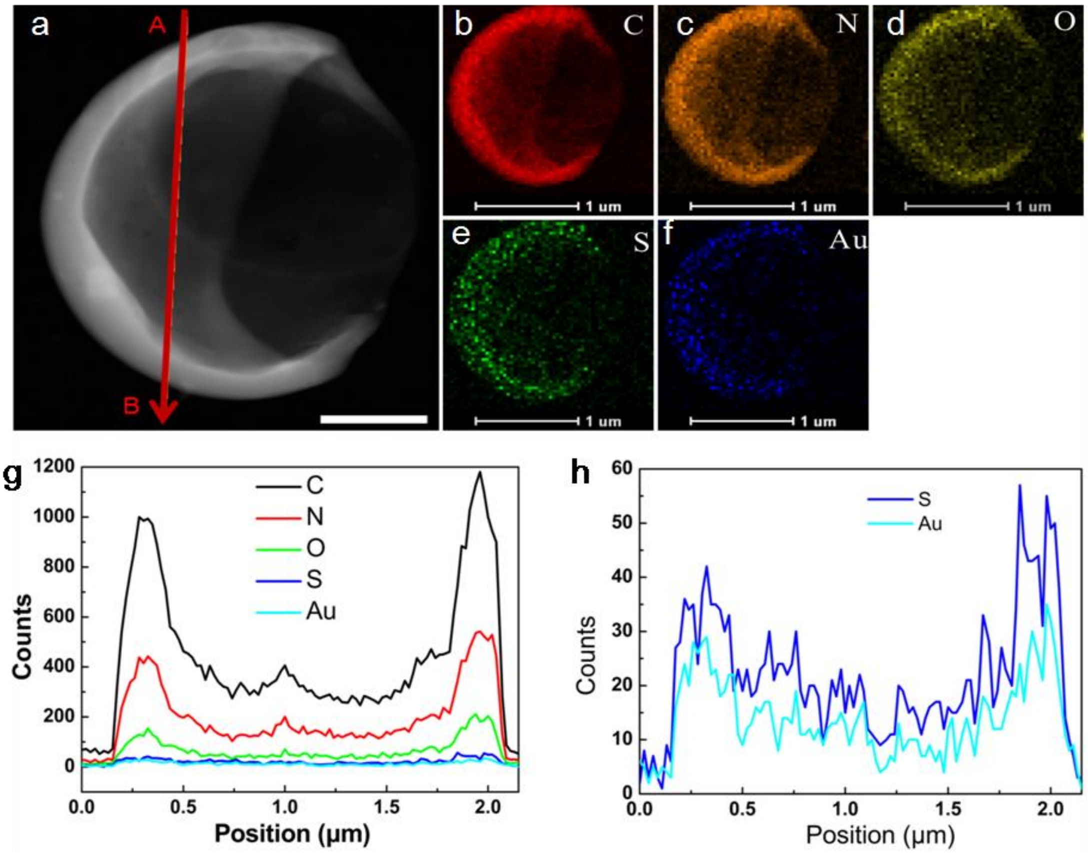

Figure S12. High resolution transmission electron microscopy (HRTEM) showing continuous and robust HPs-Au membranes (a). (b-f) the C, N, O, S and Au elemental distribution. The corresponding EDS line profile analysis and C, N, O, S and Au elemental distribution (g-h) taken from A to B in (a). Scale bars in a is 500 nm and 1μm in b-f.

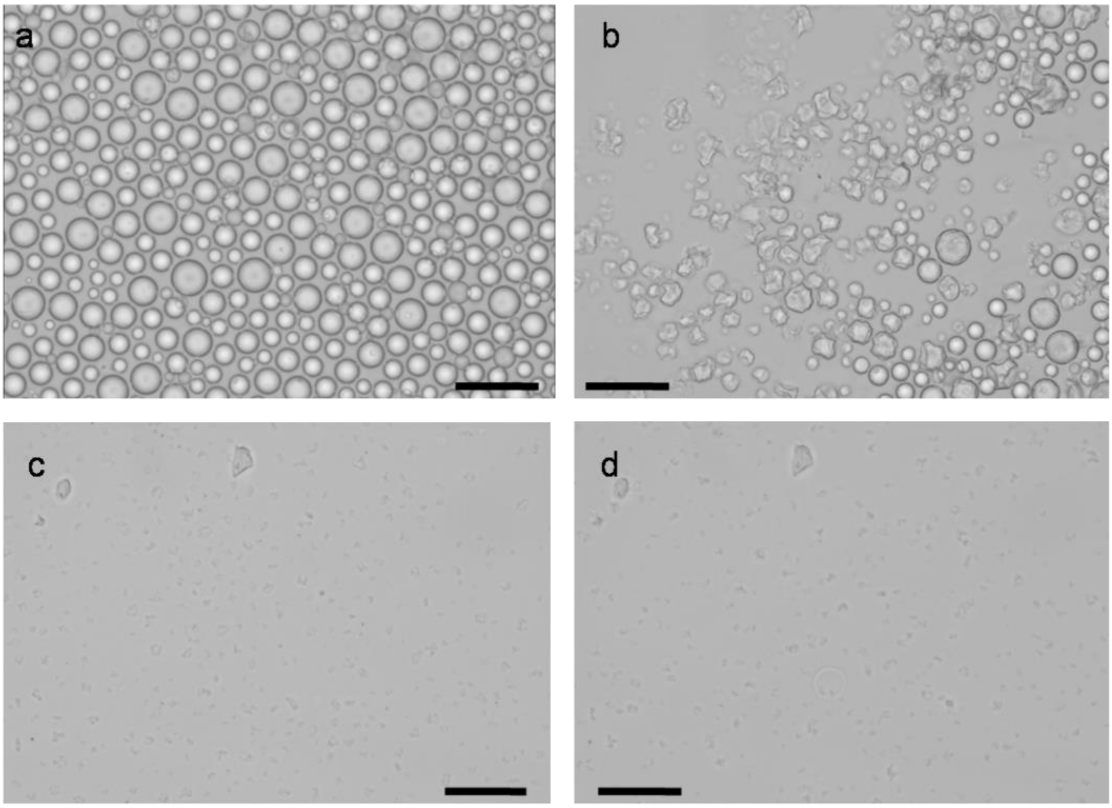

Figure S13. Optical microscope images of BSA-NH_2/PNIPAAm proteinosomes in oil (a), after 15 minutes (b), 10 hours (c) drying in air and after adding water (d), respectively. Scale bar, 50μm.

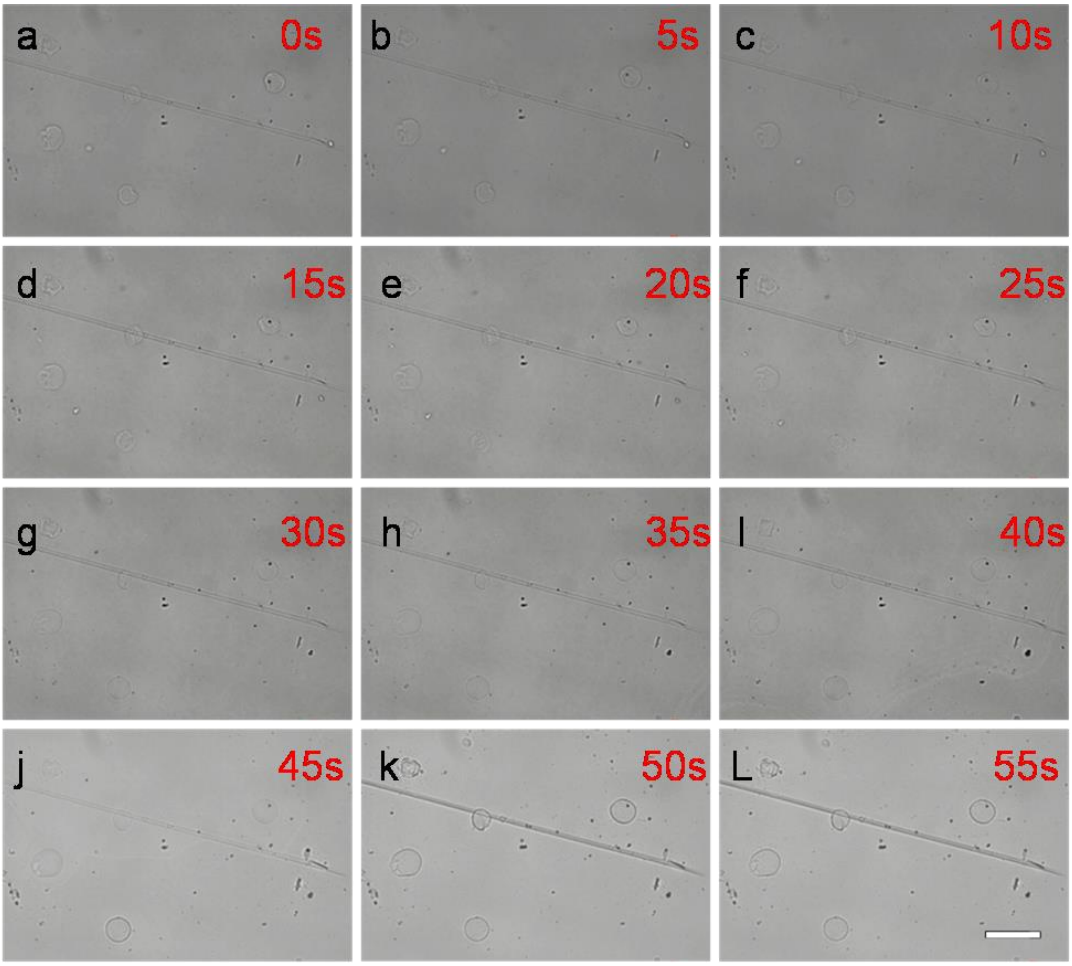

Figure S14. Optical microscopy images showing self-rehydration behavior of HPs-Au in water/water by adding water 0 and 55 seconds; Scale bar, 50μm.

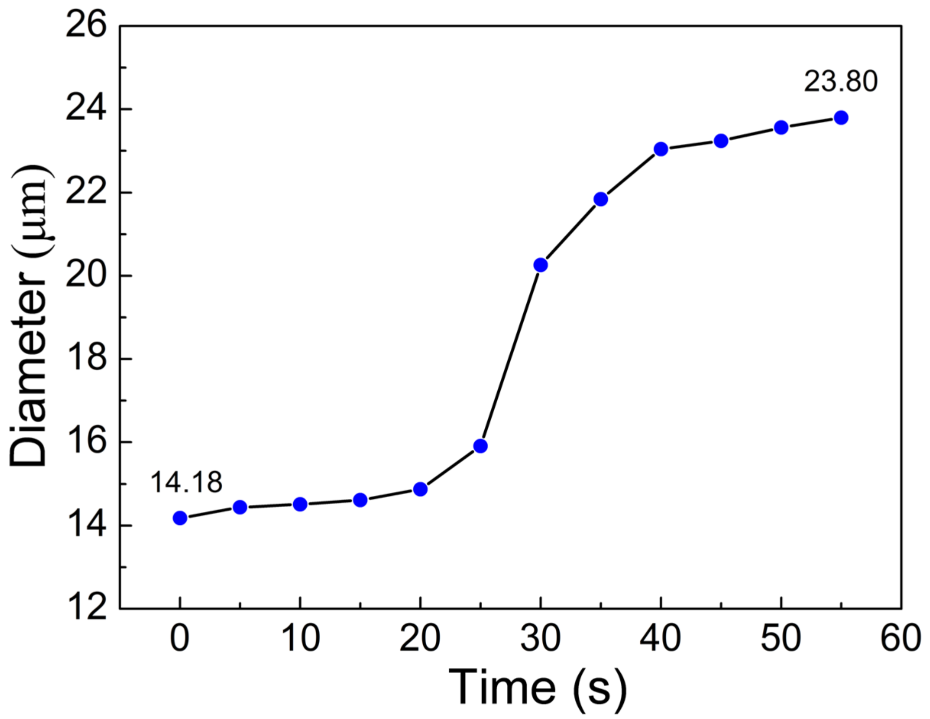

Figure S15. The changes of diameter during the process of self-rehydration behavior of HPs-Au from Figure S14.

Labelling Lipase with fluorescence dyes

Lipase (8.0 mg) was dissolved into 4.0 mL of pH 8.5 sodium carbonate buffer solution (100 mM). Then 50 μL of fluoresce in isothiocyanate DMSO solution (1.0 mg/mL) was added dropwise. The solution was stirred at room temperature for 5 h, purified by dialyzing against Milli-Q water, and freeze-dried.

Synthesis of Lipase-Au NCs and Lipase-Au NCs-PNIPAAm

Lipase (5 mg) was dissolved in deionized water (5 mL) under stirring. Chloroauric acid (0.125 mmol) was drop wisely added to the solution, after 30min incubation in 37°C, the pH of this mixture was adjusted to 12 by adding NaOH (0.125 mol) and the bottle was incubated in 37 °C oven for 12 h. Then the solution was dialyzed (dialysis tubing 8-14 kDa MWCO) extensively against Milli-Q water and freeze-dried on a Freeze-Dry System (P ≈ 10^{-4} bar for 24 h, about 5.6 mg) and stored at -20 ^{o}C.

Mercaptopyridine-activated PNIPAAm was added to a stirred solution of lipase-Au NCs (2.8 mg in 5 mL of deionized water). The mixed solution was stirred for 0.5 h, andthen purified by using a centrifugal filter (MWCO 50 kDa) to remove any unreacted PNIPAAm and salts. After freeze-drying, the lipase-Au-PNIPAAm conjugates were obtained.

Interfacial and Internal Catalysis

Interfacial Catalysis: 0.06 mL of aqueous BSA-AuNCs-PNIPAAm (10 mg/mL, pH

8.5, sodium carbonate buffer) and lipase-Au NCs-PNIPAAm (1.6 mg/mL, pH 8.5, sodium carbonate buffer) were mixed with 1.0 mL of the oil. After shaking 2 minutes, 0.2 mL of 4-methylumbelliferyl butyrate (10 mg/mL, 2-ethyl-1-hexanol) was adding into emulsion. Every time (0, 1, 2, 3, 4, 5, 7 and 10 min) 0.1 mL of emulsion was added to 0.4mL ethanol and used for fluorescence intensity test.

Internal Catalysis：0.06 mL of aqueous BSA-AuNCs-PNIPAAm(10 mg/mL, pH 8.5, sodium carbonate buffer) and Lipase (1 mg/mL, pH 8.5, sodium carbonate buffer) were mixed with 1.0 mL of the oil. After shaking 2 min, 0.2 mL of 4-methylumbelliferyl butyrate (10 mg/mL, 2-ethyl-1-hexanol), was adding into emulsion. Every time (0, 1, 2, 3, 4, 5, 7 and 10 min) 0.1 mL of emulsion was added to 0.4 mL of ethanol and used for fluorescence intensity test.

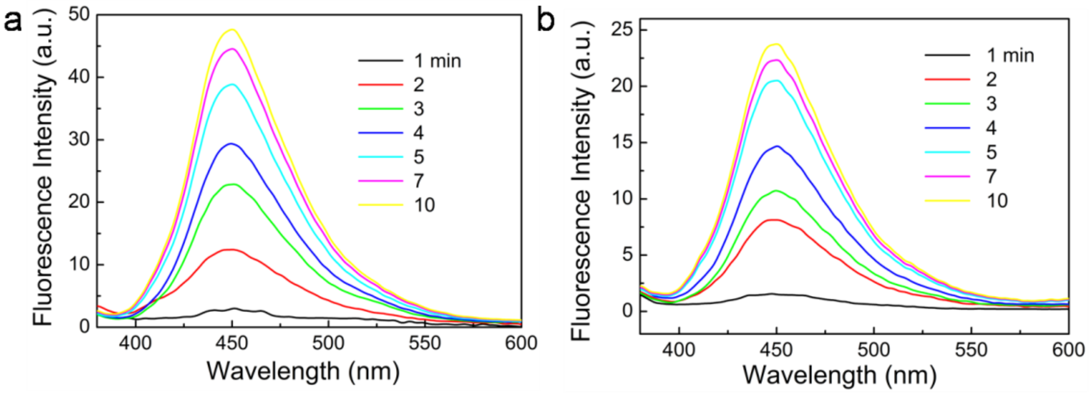

Figure S16. The effect of time on the fluorescence spectra of 4-methylumbelliferone by interfacial (a) and internal (b) catalysis at 1, 2, 3, 4, 5, 7 and 10min,using an excitation wavelength of 365 nm.

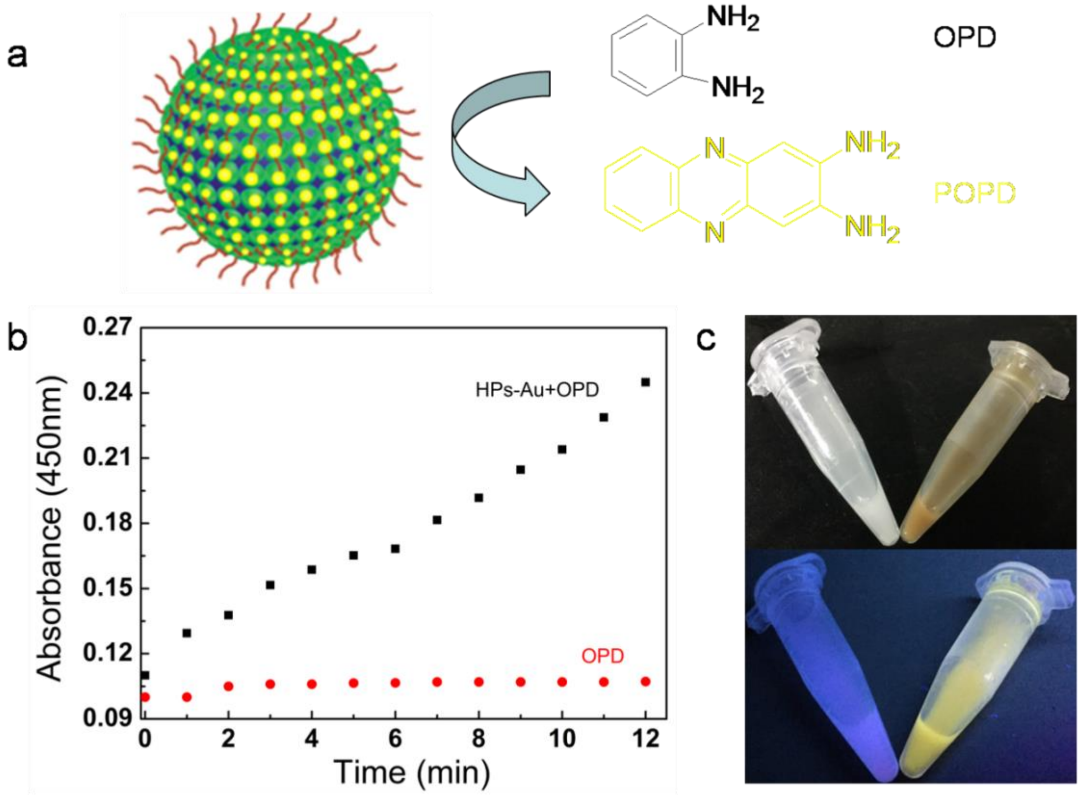

Figure S17. (a) Schematic representation of interfacial catalysis; the original product having weakly fluorescent was dissolved into oil phase and the catalysis production (POPD) having strongly yellow fluorescent. (b) The time-dependent absorbance changes at 450 nm of OPD with or without 113 μg/mL HPs-Au microcapsules. (c) Photographs of HPs-Au microcapsules without/with OPD (from left to right) under (up) visible and (bottom) UV light.

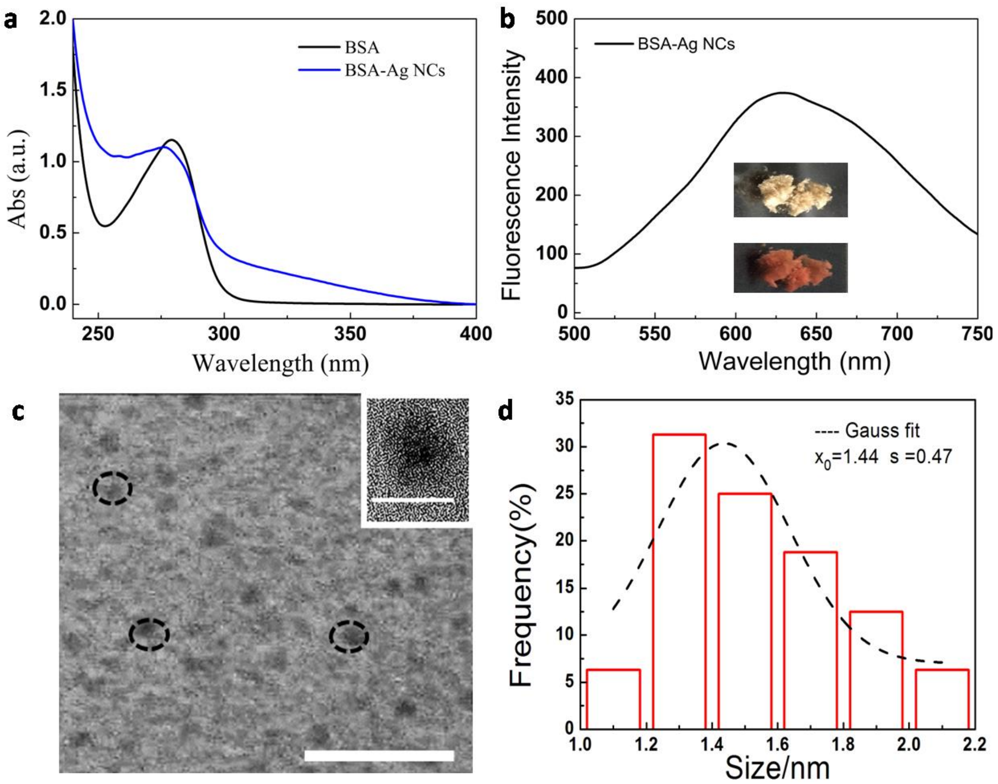

Figure S18. UV-vis absorption (a) and emission (b) spectra of the as prepared BSA-AgNCs [The inset Photographs of BSA-Ag NCs powder under (top) visibleand (bottom) UV light]. TEM images of BSA-AgNCs (c). Particle size distributions of AgNCs (d).

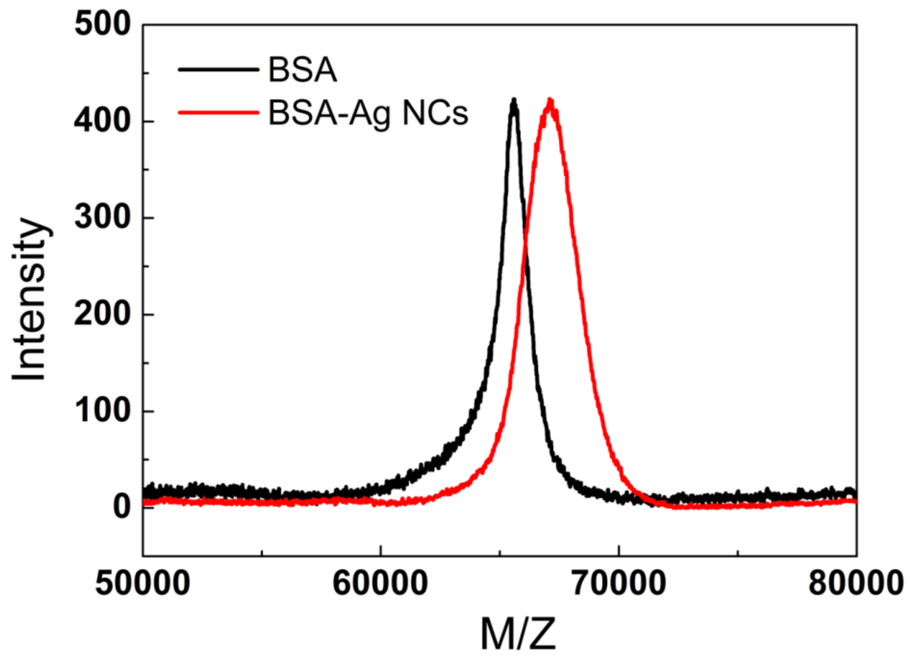

Figure S19. MALDI-TOF mass spectra of BSA (black) and BSA-AgNCs (red).

As can be seen from **Figure S19**, BSA shows a broad mass peak around m/z **65.5 KDa** but mass peak corresponding to BSA-Ag NCs is shifted toward right, appearing at **67.1KDa**. The peak difference between BSA and BSA-Ag NCs is of the order of **1.6kDa**. This difference corresponds to **15** atoms of silver.

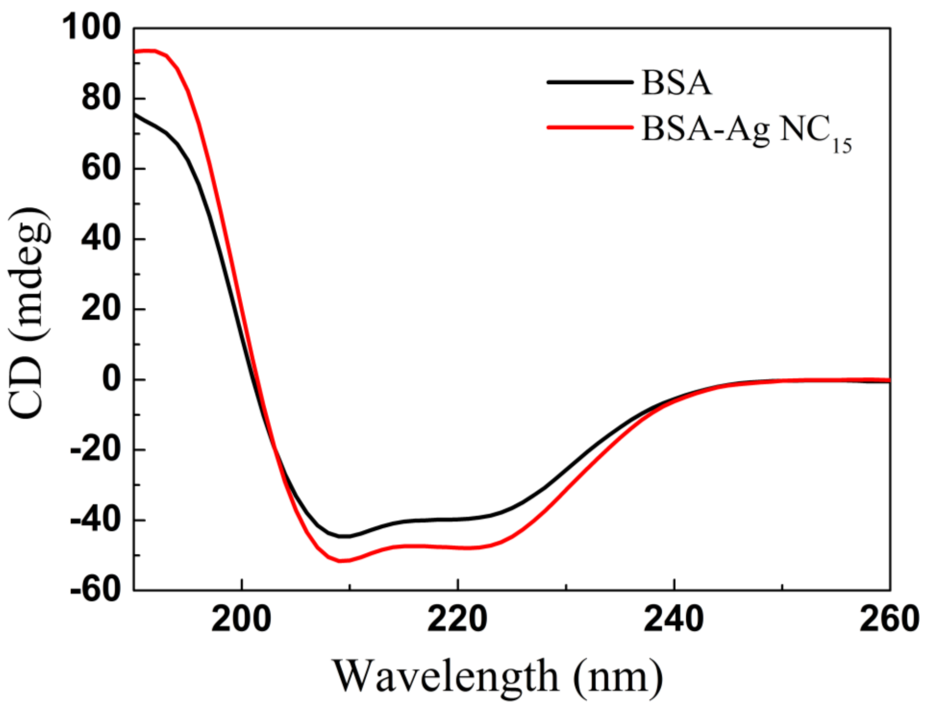

Figure S20. CD spectra for BSA and BSA-Ag NC_{15} dispersed in aqueous solution at room temperature.

Table S3. Secondary structure content in various BSA and BSA-Ag NC_{15} constructs at 25 ℃.

	α helix	β strand
BSA	67.29%	10.06%
BSA-Ag NC_{15}	67.10%	10.26%

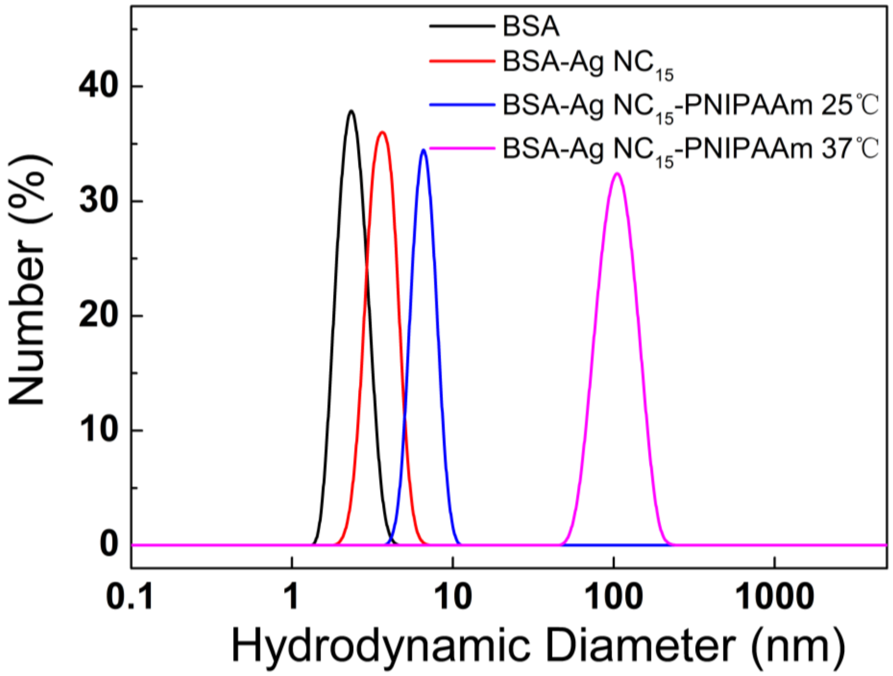

Figure S21. DLS profiles for aqueous BSA, BSA-AgNC$_{15}$ and BSA-AgNC$_{15}$-PNIPAAm conjugates recorded at 25 ℃. Profile for aqueous BSA- AgNC$_{15}$-PNIPAAm at 37 ℃ is also shown.

DLS study, there was ca. 2.95 nm increase on the mean hydrodynamic diameters after conjugating PNIPAAm compared with that of BSA-Ag NC$_{15}$ at 25 ℃ (3.67 and 6.62 nm for BSA-AgNC$_{15}$ and BSA-AgNC$_{15}$-PNIPAAm, respectively); and such size difference could be further varied when the measurement temperature was increased to 37 °C. The mean hydrodynamic diameters of the BSA-AgNC$_{15}$-PNIPAAm conjugate increased to be 102.73 nm which was due to the fact that when it was above the lower critical solution temperature of PNIPAAm (ca. 32 °C), the increased hydrophobicity of PNIPAAm would induce the aggregation of the conjugates.

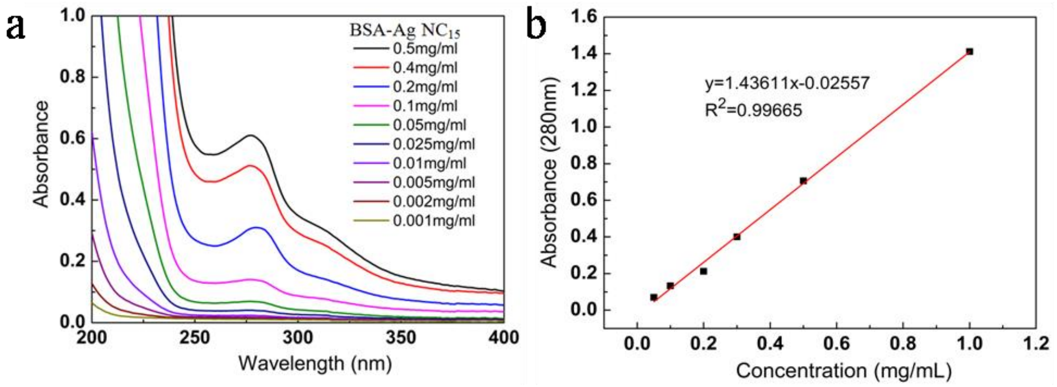

Figure S22: (a) UV-vis spectra of native BSA-Ag NC_{15} at different concentrations from 0.05 to 0.5 mg/mL in aqueous solution. (b) Calibration curve for native BSA-Ag NC_{15} determined by plotting the UV-vis absorbance at 280 nm against concentration. The calibration was used to determine the number of PNIPAAm chains per protein molecule in the BSA-AgNC_{15}-PNIPAAmnano-conjugates (see **Table S4** below)

Table S4. PNIPAAm : BSA-$AgNC_{15}$ stoichiometry in BSA-$AgNC_{15}$-PNIPAAm conjugates based on UV-vis spectroscopic analysis of three different sample concentrations. From the UV spectra calculation, on average, there were 1.35 conjugated PNIPAAm per BSA-$AgNC_{15}$.

	Concentration (mg/mL)	A(280)	PNIPAAm Content (chains perBSA-$AgNC_{15}$-PNIPAAm)
Test 1	0.05	0.05327	1.35
Test 2	0.10	0.09086	1.37
Test 3	0.50	0.54748	1.33

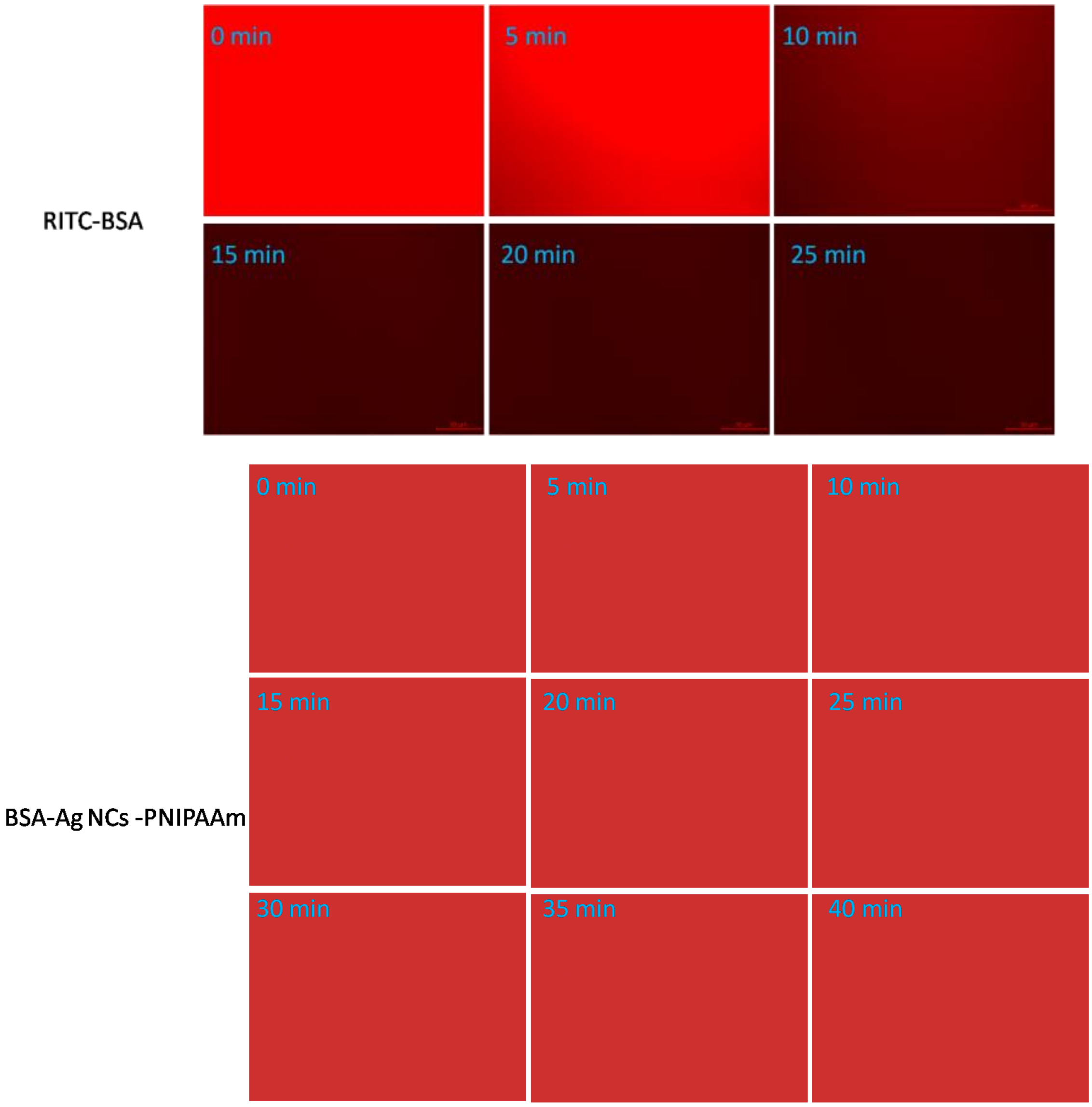

Figure S23: Photostability of BSA-Ag NC_{15}-PNIPAAm (1 mg/mL) compared to Rhodamine B isothiocyanate labeled BSA (RITC-BSA) under continual excitation on fluorescence microscope

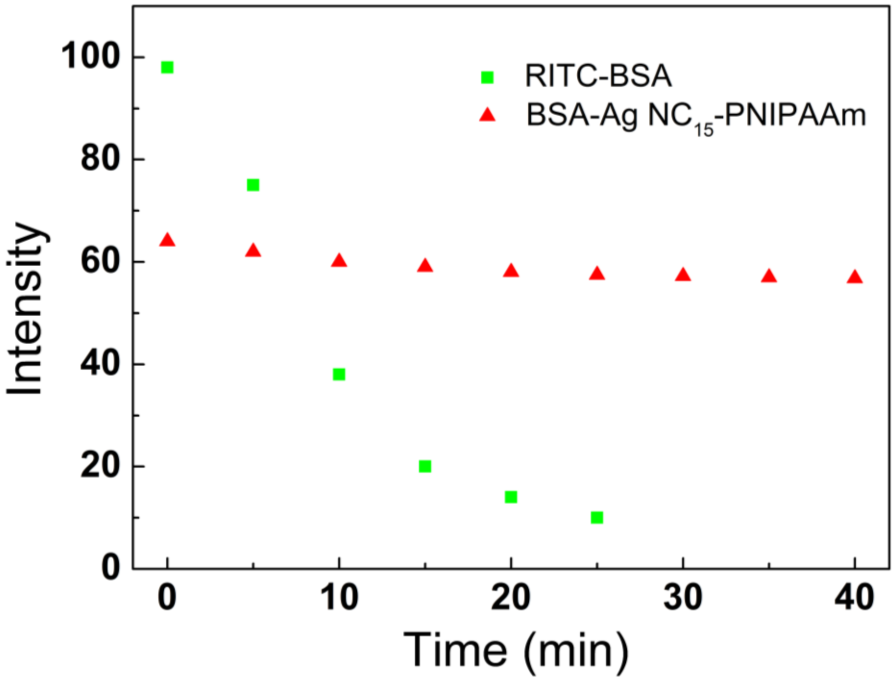

Figure S24: Data derived from Figure S23 quantified by ImageJ software.

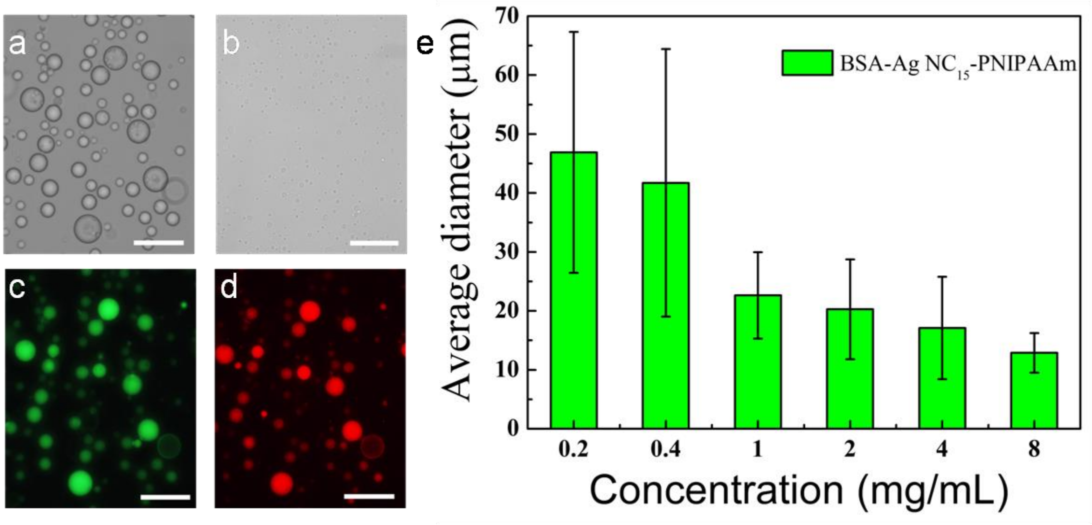

Figure S25. Optical microscope images in oil phase (**a** and **b**) and after partial drying in air (**c and d**) fluorescence microscope image of BSA-$AgNC_{15}$-PNIPAAm hybrid microcapsulesin aqueous solution; the green fluorescence originates from encapsulating fluoresce in isothiocyanate labeled dextran (FITC-Dextran MW 500 kDa); the red fluorescence originates from emission of $AgNC_{15}$. (**e**) Plot showing mean size (bars) and s.d. (lines on bars) of hybrid microcapsules dispersed in oil and prepared at different concentrations of BSA-$AgNC_{15}$-PNIPAAm between 0.2-8.0 mg/mL.

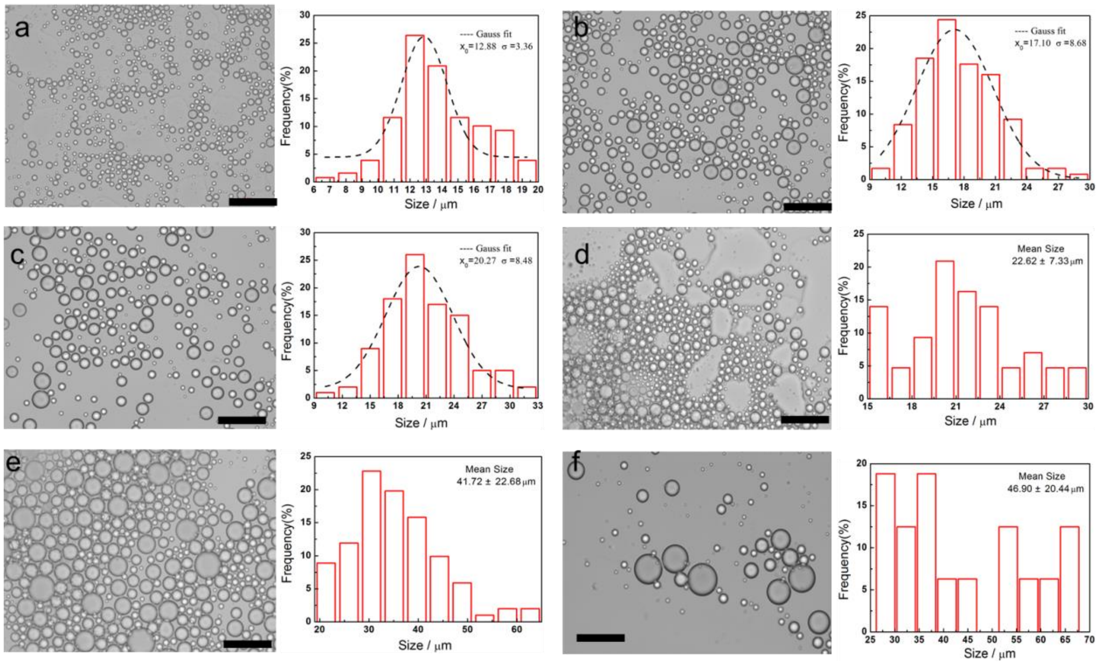

Figure S26. (a-f) Particle size distribution of hybrid microcapsules prepared atdifferent concentrations of BSA-AgNC$_{15}$-PNIPAAm (8.0, 4.0, 2.0, 1.0, 0.4 and 0.2 mg/mL, respectively). Median values and standard deviations were calculated by fitting Gaussians to the histograms in a-c. All scare bars are 50 μm

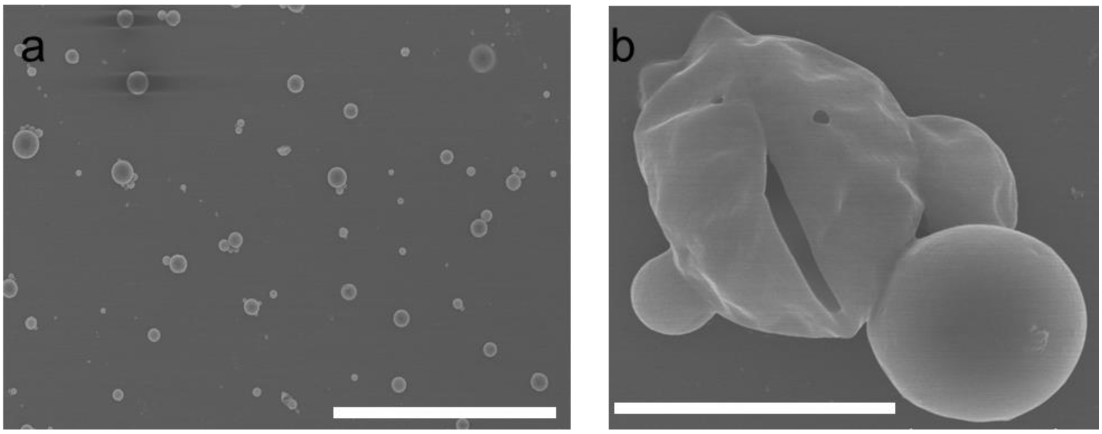

FigureS27. Scanning electron microscope (SEM) showing a hollow and stabilized structure of HPs-Ag. Scale bars in a, b are 100 and 10μm, respectively.

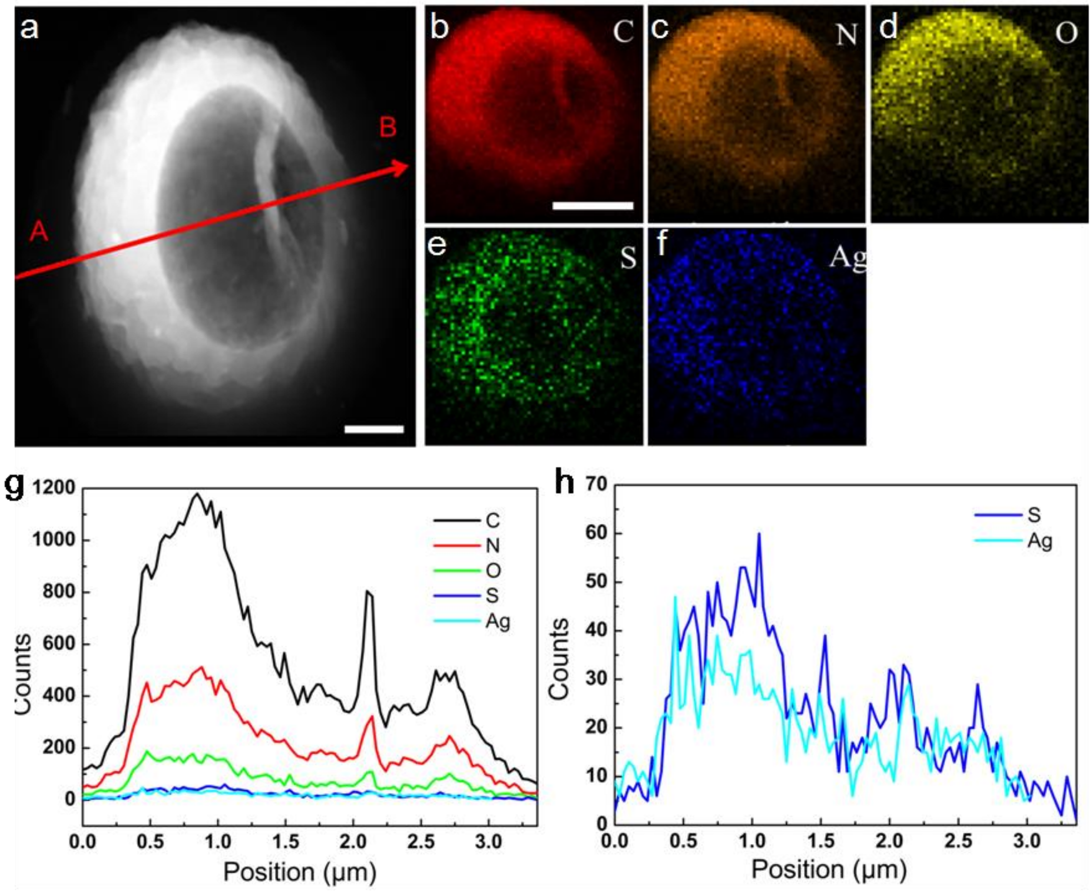

Figure S28. High resolution transmission electron microscopy (HRTEM) showing continuous and robust HPs-Ag membranes (**a**). (**b-f**) the C, N, O, S and Ag elemental distribution. The corresponding EDS line profile analysis and C, N,O,S and Ag elemental distribution (**g-h**) taken from A to B in (a). Scale bars in a is 500 nm and 1μm in b-f.

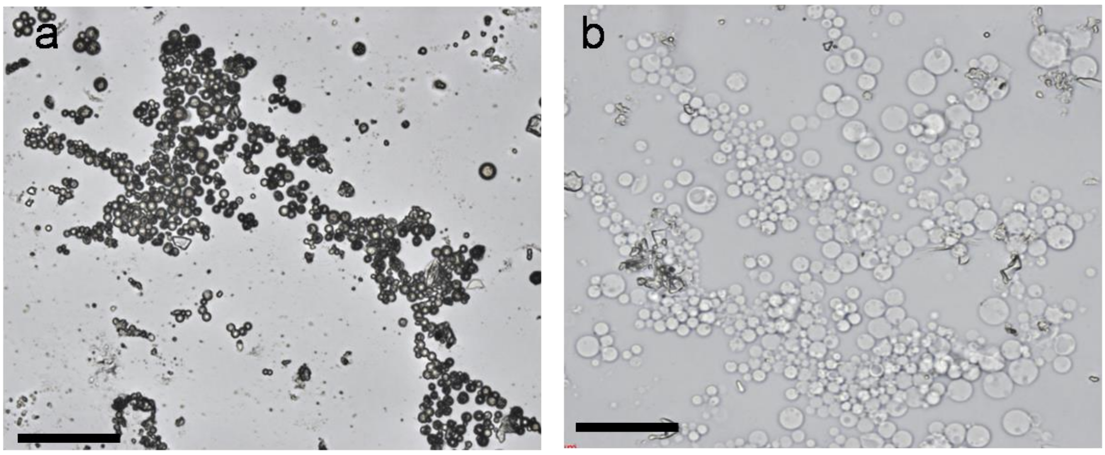

Figure S29. Optical microscopy images showing self-rehydration behavior of HPs-Ag by adding water 0 and 10 seconds; Scale bar=50μm (Movie S2).

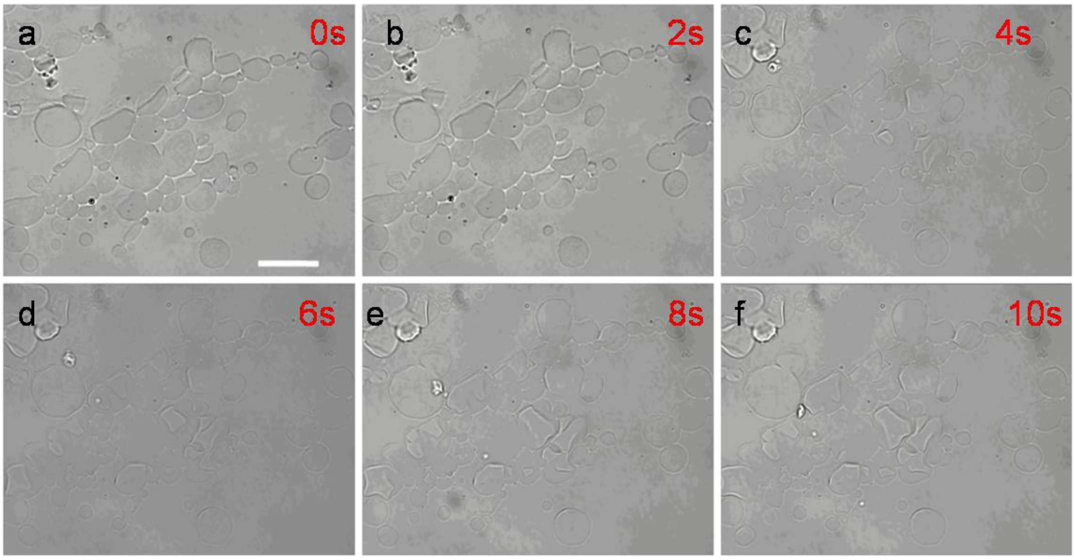

Figure S30. Optical microscopy images showing self-rehydration behavior of HPs-Ag in water/water by adding water 0 and 90 seconds; Scale bar, 50μm.

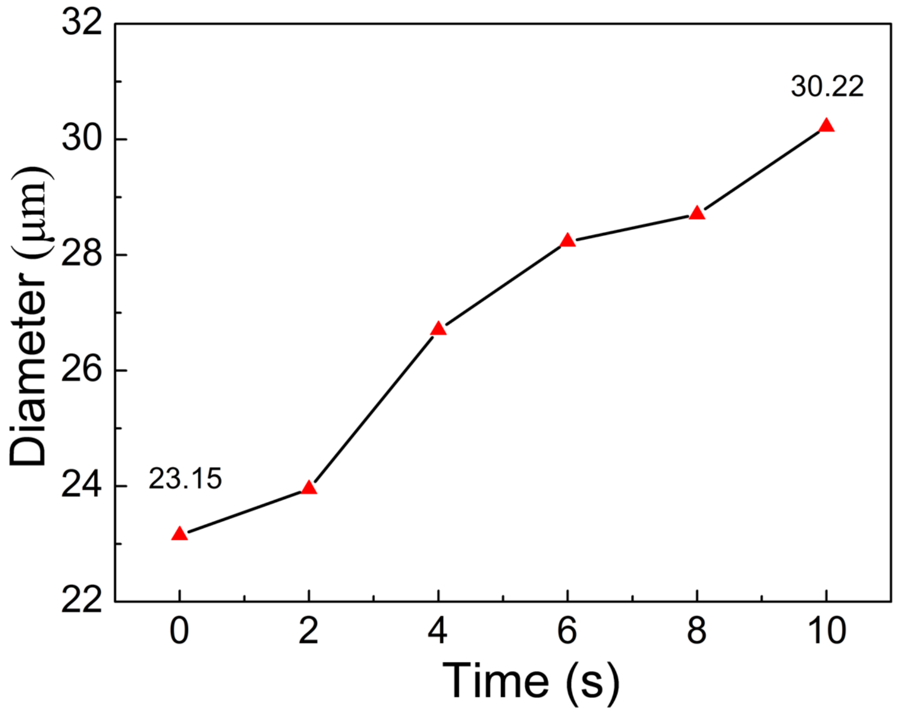

Figure S31. The changes of diameter during the process of self-rehydration behavior of HPs-Ag from Figure S30.

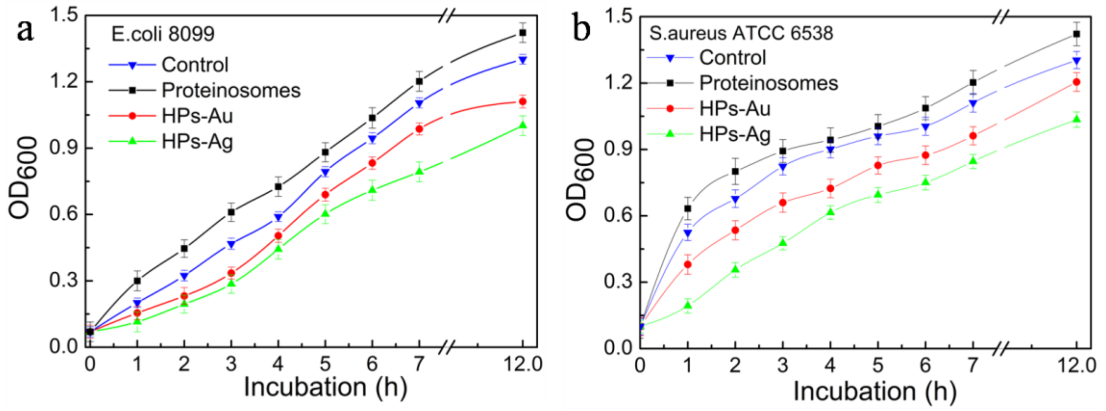

Figure S32. Optical density curves indicating bacterial growth in LB Lennox liquid medium, pH=6.0. (a) E. coli, proteinosomes, HPs-Au, HPs-Ag(b) S. aureus, proteinosomes, HPs-Au, HPs-Ag. Each symbol indicates the means ± standard errors for three observations.

OD=600, the number of E. coli is around 10^8, so, the result of E. coli, proteinosomes, HPs-Au, HPs-Ag are (1.302, 1.422, 1.111, 1.002)*10^8. After adding HPs-Au and HPs-Ag, the numbers of bacteria cells killed are 1.91*10^7 and 3.00*10^7, respectively.

OD=600, the number of S. aureus is around 10^8, so, the result of S. aureus, proteinosomes, HPs-Au, HPs-Ag are (1.304, 1.425, 1.205, 1.035)*10^8. After adding HPs-Au and HPs-Ag, the numbers of bacteria cells killed are 9.9*10^6 and 2.69*10^7, respectively.

SI Video 1. The deflated microcapsules (HPs-Au) produced on drying could be reinflated to the spherical morphology within 120s by adding water. Scale bar = 50 μm.

SI Video 2. The deflated microcapsules (HPs-Ag) produced on drying could be reinflated to the spherical morphology within 90s by adding water. Scale bar = 50 μm.

Reference:

[1] J. Xie, Y. Zheng, J .Y. Ying, *J. Am. Chem. Soc*. **2009**, *131*, 888.

[2] A. Baksi, P. L. Xavier, K. Chaudhari, N. Goswami, S. K. Pal, T. Pradeep, *Nanoscale*, **2013**, *5*, 2009.

[3] A. Mathew, P. R. Sajanlal, T. Pradeep, *J. Mater. Chem.* **2011**, *21*, 11205.

[4] X. Huang, M. Li, D. C. Green, S. Williams, A. J. Patil, S. Mann, *Nat. Commun.* **2013**, *4*, 2239.
